# Cooperative Roles of Class IA PI3K Isoforms in Translocation-Related Sarcoma Cell Survival and Proliferation

**DOI:** 10.1158/2767-9764.CRC-25-0787

**Published:** 2026-04-29

**Authors:** Sho Isoyama, Naomi Tamaki, Yutaka Noguchi, Hiroki Shinchi, Takeshi Suzuki, Makoto Hirata, Kotoe Katayama, Hidewaki Nakagawa, Koichi Matsuda, Shin-ichi Yaguchi, Koji Ueda, Shingo Dan

**Affiliations:** 1 Division of Molecular Pharmacology, Cancer Chemotherapy Center, Japanese Foundation for Cancer Research, Tokyo, Japan.; 2 Cancer Proteomics Group, Cancer Precision Medicine Center, Japanese Foundation for Cancer Research, Tokyo, Japan.; 3 Division of Functional Genomics, Cancer Research Institute, Kanazawa University, Kanazawa, Japan.; 4 Laboratory of Genome Technology, Institute of Medical Science, The University of Tokyo, Tokyo, Japan.; 5 Department of Genetic Medicine and Services, National Cancer Center Hospital, Tokyo, Japan.; 6 Laboratory of Sequence Analysis, Human Genome Center, Institute of Medical Science, The University of Tokyo, Tokyo, Japan.; 7 Laboratory for Cancer Genomics, RIKEN Center for Integrative Medical Sciences, Yokohama, Japan.; 8 Laboratory of Clinical Genome Sequencing, Graduate School of Frontier Sciences, The University of Tokyo, Tokyo, Japan.

## Abstract

Among class I phosphatidylinositol 3-kinase (PI3K) isoforms, the isoform that predominantly regulates proliferation and survival in cancer dependent on PI3K signaling differs according to the genetic background and lineage of origin. We previously reported that translocation-related sarcomas (TRS) such as synovial sarcoma and Ewing sarcoma are highly susceptible to pan-class I PI3K inhibitors, but the dominant isoform remains unclear. To address this issue, we examined the roles of each PI3K isoform in TRS cells. Neither class I PI3K isoforms nor PI3K-related genes in sarcoma cell lines, including TRS, exhibited common mutations. Selective inhibition of PI3Kα moderately suppressed Akt/mTOR signaling, leading to growth inhibition and apoptosis, whereas inhibiting PI3Kβ or PI3Kδ alone had no effect. Interestingly, inhibition of PI3Kα together with PI3Kβ and/or PI3Kδ significantly enhanced apoptosis induction versus PI3Kα inhibition alone. In contrast, carcinoma cell lines did not undergo apoptosis upon PI3K inhibition, except for *PIK3CA*-mutated cell lines. In those cell lines, PI3Kα inhibition alone significantly induced apoptosis with no enhancement in simultaneous inhibition of PI3K isoforms. Mechanistically, whereas PI3Kα primarily mediated Akt/mTOR signaling in TRS cells, PI3Kβ and PI3Kδ compensated for Akt/mTOR signaling when PI3Kα was inhibited. Simultaneous inhibition of PI3Kα, PI3Kβ, and PI3Kδ was more potent than individual inhibition against TRS cells in a mouse xenograft model. These findings suggest that PI3Kα is the dominant isoform, whereas PI3Kβ and PI3Kδ cooperate with PI3Kα in cell survival, which seems to be a characteristic feature of TRS cells. Thus, triple-isoform inhibition might represent an effective therapy for TRSs.

**Significance:**

PI3Kα is the dominant isoform regulating cell survival, whereas PI3Kβ and PI3Kδ complement PI3Kα, suggesting that coinhibition of class IA PI3K isoforms could be a potential therapeutic strategy for TRSs.

## Introduction

Sarcomas are rare malignant tumors arising from mesenchymal cells of connective tissues, and more than 170 different tissue subtypes have been reported (1). It is estimated that more than 17,000 new cases of sarcoma were diagnosed in the United States in 2025, resulting in more than 7,000 deaths despite aggressive treatment, including surgery, radiation, and chemotherapy (2). Approximately 20% of sarcomas are classified as translocation-related sarcomas (TRS), which harbor oncogenic fusion genes generated by chromosome translocation (3). Most fusion genes in TRSs, such as *SS18::SSX*, *EWSR1::FLI1*, and *PAX3::FOXO1*, which are responsible for synovial sarcoma (SS), Ewing sarcoma, and alveolar rhabdomyosarcoma (ARMS), respectively, drive oncogenesis through their broad gene transcriptional regulation (4–7). However, these fusion gene products are challenging targets for molecular therapies because these proteins lack surface pockets suitable for binding by small molecules (3, 8). Effective therapeutic targets for these TRSs beyond the fusion gene products have not yet been identified.

Phosphatidylinositol 3-kinase (PI3K) is classified into classes I to III based on its substrate specificity and amino acid sequence similarity. Class I PI3K, the most studied class, is further divided into class IA, consisting of PI3Kα, PI3Kβ, and PI3Kδ, and class IB consisting solely of PI3Kγ. These class I PI3K isoforms form a heterodimer consisting of catalytic (p110α, p110β, p110δ, and p110γ) and regulatory (p85, p55, and p101) subunits (9). Class I PI3K signaling is frequently activated in cancer cells by activating genetic mutations or gene amplification of p110α (encoded by *PIK3CA*) and upstream receptor tyrosine kinases (RTK) and loss of function of PTEN, a phosphatase that inversely reacts with class I PI3K, contributing to cell survival, proliferation, and migration (9). These findings prompted the development of many PI3K inhibitors; however, results from initial clinical trials testing the use of pan-PI3K inhibitors for solid tumors have been disappointing (10). Subsequently, several class I PI3K isoform–specific inhibitors were developed, among which the PI3Kα-specific inhibitor alpelisib was approved by the US Food and Drug Administration (FDA) for patients with *PIK3CA*-mutated HR^+^HER2^−^ breast cancer. PI3Kβ-specific inhibitors such as TGX-221 were developed primarily for prostate cancers and brain tumors with *PTEN* loss-of-function mutations, although none of the PI3Kβ-specific inhibitors has been approved for patients with cancer in the United States. Because PI3Kδ is expressed primarily in blood cells and it plays a fundamental role in several processes, particularly downstream signaling of B-cell antigen receptor (BCR), several inhibitors of PI3Kδ have been developed, and some of them, including idelalisib, have been approved for B-cell malignancies such as chronic lymphocytic leukemia (CLL), small lymphocytic lymphoma (SLL), and follicular lymphoma (FL; ref. 11). Thus, *PIK3CA*-mutated cancers, PTEN-deficient cancers, and B-cell malignancies are dependent on PI3K signaling, with PI3Kα, PI3Kβ, and PI3Kδ functioning as the dominant isoform, respectively. These findings indicate that the dominant PI3K isoform differs according to the genetic background and lineage of the cancer dependent on PI3K signaling.

Although the clinical efficacy of PI3K inhibitors for patients with sarcoma remains unclear, we previously reported that ZSTK474, a pan-PI3K inhibitor developed by our group, produced long-term disease stability in three of four patients with sarcoma enrolled in a phase Ib clinical trial of patients with solid tumors (NCT01280487 and NCT01682473; refs. 12, 13). To explore sarcoma subtypes sensitive to PI3K inhibitors, we previously established a sarcoma panel consisting of cell lines and patient-derived cells (PDC) from various sarcoma origins and found that pan-PI3K inhibitors exhibit significant antitumor effects against cells derived from TRSs such as SS, Ewing sarcoma, and ARMS (14, 15). However, the class I PI3K isoform that plays a fundamental role in sarcomas, especially TRSs, remains unclear.

In this study, we investigated the roles of individual class I PI3K isoforms using isoform-specific inhibitors, as well as gene knockdown with siRNAs and gene knockout via the CRISPR/Cas9 system, in signaling, proliferation, and viability in TRS cells. We demonstrated that PI3Kα plays a primary role in the transduction of PI3K signaling, as well as in proliferation, survival, and cell-cycle progression in TRS cells. By contrast, PI3Kβ and PI3Kδ have small but significant effects, which can compensate for the effects of PI3Kα and sustain the robustness of TRS cells in tumor proliferation and survival. Our results provide proof that inhibition of all three isoforms by pan-class I PI3K inhibitors is effective in the treatment of TRSs.

## Materials and Methods

### Cell lines and cell culture

The cell lines used in this study were obtained as described previously (14, 15) and authenticated by short tandem repeat analysis. For all TRS cell lines used in this study, we confirmed the presence of the expected fusion transcripts by inspection of RNA sequencing (RNA-seq) data using Integrative Genomics Viewer, demonstrating that sequencing reads were split at the appropriate exon boundaries of the fusion genes. *Mycoplasma* testing for all cell lines used in animal models was performed by the Central Institute for Experimental Medicine and Life Science prior to use in mice, and no *Mycoplasma* contamination was detected. The remaining cell lines were not tested for *Mycoplasma* contamination. All cell lines were maintained in RPMI-1640 medium supplemented with 5% (v/v) fetal bovine serum and 1 μg/mL kanamycin at 37°C in humidified air containing 5% CO_2_.

### Drugs

Alpelisib, TGX-221, and idelalisib for *in vitro* experiments were purchased from Selleck Chemicals. Alpelisib, AZD6482, and idelalisib for *in vivo* experiments were purchased from MedChemExpress. ZSTK474 was provided by Ohara Pharmaceutical, Co., Ltd. These compounds were dissolved in DMSO (Sigma-Aldrich) for *in vitro* experiments.

### RNA-seq

Total RNA was extracted from each cell line using an RNeasy Mini Kit (Qiagen). After a quality control step with a 2100 bioanalyzer (Agilent Technologies), the library was constructed using a TruSeq Stranded mRNA Prep Kit (Illumina) according to the manufacturer’s instructions and subjected to sequencing using a HiSeq2000 or HiSeq2500 sequencer (Illumina). The RNA-seq FASTQ files of SU-DHL-4 cells (GSM6285348), SU-DHL-6 cells (GSM6285350, RRID: CVCL_2206), SS (GSM8378222, GSM8378226, GSM8378227, GSM8378248, and GSM8378286), Ewing sarcoma (GSM8332627-GSM8332631), breast cancer (GSM8141778-GSM8141782), and diffuse large B-cell lymphoma (DLBCL; GSM5402206-GSM5402210) were obtained from Gene Expression Omnibus (GEO, RRID: SCR_005012) database. Low-quality bases and sequence adapters were removed using FASTP (version 0.23.4, RRID: SCR_016962). The cleaned paired-end reads were aligned to the reference human genome (GRCh38/hg38) using STAR (version 2.7.11a, RRID: SCR_004463). The resulting SAM files were transformed into BAM files using SAMtools (version 1.18, RRID: SCR_002105). Gene expression was quantified using HTseq (version 2.0.4, RRID: SCR_005514). Raw mapped read counts were normalized to transcripts per million.

### Whole-exome sequencing

Genomic DNA was extracted from each cell line using DNeasy Blood & Tissue Kit (Qiagen). The exon DNA library was prepared using SureSelect XT Human All Exon V7 Kit (Agilent Technologies) following the manufacturer’s instructions and sequenced using the HiSeq2000 or HiSeq2500 sequencer. Subsequently, low-quality bases and sequence adapters were removed using Cutadapt v4.1 (RRID: SCR_011841). Cleaned reads were aligned to the reference human genome (GRCh38/hg38) using the Burrows–Wheeler Aligner (v0.7.17). Duplicated reads were removed using the Genomic Analysis Toolkit (GATK v4.3.0.0, RRID: SCR_001876). Single-nucleotide variants and indel variants were called by HaplotypeCaller of GATK v.4.3.0.0. Called variants with low quality (variant depth <10, genotype quality <50, and variant allele frequency <0.1) and those not listed in the Catalogue of Somatic Mutation in Cancer database were removed.

### Immunoblotting

Immunoblotting was performed on cell extracts as described previously (16) using the primary antibodies listed in Supplementary Table S1. Briefly, cells were washed with PBS and lysed with 10 mmol/L Tris-HCl buffer at pH 7.4 containing 50 mmol/L NaCl, 50 mmol/L NaF, 30 mmol/L sodium pyrophosphate, 50 mmol/L Na_3_VO_4_, 5 mmol/L EDTA, 100 KIU/mL aprotinin, 1 mmol/L phenylmethylsulfonyl fluoride, 0.5% Nonidet P-40, and 0.1% sodium dodecyl sulfate. Xenograft tumors were homogenized in the lysis buffer with Precellys 24 (Bertin Technologies). Then, 10 μg of lysate per lane were resolved by SDS-PAGE and transferred onto a nitrocellulose membrane. After blocking with Intercept Blocking Buffer (LI-COR), the membrane was incubated with primary antibodies at 4°C overnight, followed by incubation with secondary antibody labeled with Alexa Fluor 680 or Alexa Fluor 800 (Supplementary Table S1) for 1 hour at room temperature. The signals were detected using the Odyssey Infrared Imaging System (LI-COR).

### Cell growth assay

Drug efficacy was assessed as changes in the total cellular protein after drug treatment using the sulforhodamine B (SRB) assay. Briefly, cells were plated in 96-well plates and cultured with the indicated drug for 48 hours. Then, 50 μL of 50% (w/v) trichloroacetic acid were added to each well, and the plates were incubated at 4°C for 1 hour. The plates were washed five times with water, and then 50 μL of 0.4% (w/v) SRB sodium salt in 1% (v/v) acetic acid were added to each well. After incubation for 10 minutes at room temperature, the plates were rinsed five times with 1% (v/v) acetic acid and then allowed to air-dry completely at room temperature. The dried SRB in each well was dissolved in 150 μL of 10 mmol/L Tris base solution, and the absorbance at 525 nm was measured using a microplate reader (Benchmark Plus, Bio-Rad Laboratories). The drug concentration required to reduce the net protein increase by 50% [50% growth inhibition (GI_50_)] was calculated as described previously (17).

### Transfection of siRNAs

Specific siRNAs against *PIK3CA* (s10520 and s10521), *PIK3CB* (s10524 and s10525), and *PIK3CD* (s10529 and s10530) and negative control siRNA (4390843) were purchased from Thermo Fisher Scientific. Cells were plated in six-well plates and transfected with 44 nmol/L siRNAs using RNAiMAX (Thermo Fisher Scientific) according to the manufacturer’s instructions. The transfected cells were then cultured for 72 hours and lysed with lysis buffer for immunoblotting. In the combination experiment with siRNAs and drugs, cells were transfected with each siRNA, and after 24 hours, the cells were cultured with the indicated drug for another 48 hours and lysed with lysis buffer for immunoblotting.

### Fluorescence time-lapse imaging using SiR-DNA and SYTOX Green

To assess the effects of PI3K inhibitors on cell proliferation and cell death, fluorescence time-lapse imaging was performed. Cells were plated in 96-well or 384-well black plates (Revvity) with SiR-DNA (Cytoskeleton, Inc.) at 500 nmol/L and SYTOX Green (Thermo Fisher Scientific) at 30 nmol/L. At 24 hours after cell plating, the indicated drugs were added to each well, and then fluorescent images of the stained cells were obtained every hour with a ×10 objective using a live-cell high-throughput imaging system (Operetta CLS, Revvity). Quantitative image analysis was performed using dedicated software (Harmony 4.9, Revvity). To segment nuclei, the *Find Nuclei* building block was applied to the SiR-DNA channel, and basic morphology (i.e., area, roundness, width, and length) properties were calculated using the *Calculate Morphology Properties* building block. The *Select Population* building block was then applied to remove border objects and mis-segmented objects based on the calculated morphology properties. To detect apoptotic cells, the fragmentation index, which is the coefficient of variance (CV) of the fluorescence intensity of SiR-DNA in the region of the selected nuclei, was calculated. To detect dead cells, SYTOX Green–positive cells were detected and counted using the *Find Nuclei* building block in the SYTOX Green channel.

### Annexin V–FITC/propidium iodide staining for apoptosis detection

To measure the induction of apoptosis upon treatment with PI3K inhibitors, annexin V-FITC/PI (Miltenyi Biotec) staining was performed according to the manufacturer’s instructions. Aska-SS cells were plated in 24-well plates and cultured for 24 hours. The cells were detached with trypsin after treatment with the indicated drugs for 48 hours. After washing with 1× binding buffer, annexin V-FITC was added and incubated for 15 minutes. After the addition of propidium iodide (PI), the stained cells were analyzed on a BD FACSMelody (BD Biosciences) using FlowJo software (BD Biosciences, RRID: SCR_008520).

### Cell proliferation assay by flow cytometry

To measure cell proliferation, the CellTracer Yellow (Thermo Fisher Scientific) dilution assay (18) was performed. Cells were plated in 12-well plates and cultured for 24 hours. The cells were then labeled via incubation with 2.5 μmol/L CellTrace Yellow for 20 minutes at 37°C and cultured with the indicated drugs for 6 days. Collected cells were further stained with SYTOX AADvanced (Thermo Fisher Scientific) for 15 minutes at 4°C to detect dead cells. CellTrace Yellow dilution was assessed using BD FACSMelody and FlowJo software. SYTOX AADvanced–positive cells were excluded from the analysis.

### Cell-cycle analysis

Cell-cycle analysis was performed by flow cytometry using Vybrant DyeCycle Violet (Thermo Fisher Scientific). Cells were plated in 12-well plates and incubated for 24 hours. Cells were then cultured with the indicated drugs for 48 hours. After cells were detached and collected, 1 mL of complete medium with 1 μL of Vybrant DyeCycle Violet and SYTOX AADvanced were added, followed by incubation at 37°C for 30 minutes. The stained cells were analyzed using BD FACSmelody and FlowJo software. SYTOX AADvanced–positive cells were excluded from the analysis.

### Phosphoproteomic profiling

To explore the pathways regulated by PI3Kβ/δ, phosphoproteomic analysis was performed. Cells were plated in six-well plates and incubated for 24 hours. Alpelisib or ZSTK474 was added at 1 μmol/L to each well, followed by 1 hour of incubation. After washing with cold PBS, cells were lysed with Phase Transfer Surfactant buffer containing 12 mmol/L sodium deoxycholate, 12 mmol/L sodium lauroyl sarcosinate, 50 mmol/L Tris-Cl, 10 mmol/L tris-(2-carboxyethyl)phosphine (TCEP), 40 mmol/L chloroacetamide, and phosphatase inhibitor. After reduction with 10 mmol/L TCEP at 100°C for 10 minutes and alkylation with 25 mmol/L iodoacetamide at ambient temperature for 45 minutes, protein samples were digested with Trypsin/Lys-C Mix (Promega) at 47°C for 2 hours on S-Trap columns (ProtiFi). Subsequently, phosphopeptides were purified using a High-Select TiO2 Phosphopeptide Enrichment Kit (Thermo Fisher Scientific) according to the manufacturer’s instruction. The resulting peptides were extracted from gel fragments and analyzed using Orbitrap Fusion Lumos mass spectrometer (Thermo Fisher Scientific) combined with UltiMate 3000 RSLC nano-flow HPLC (Thermo Fisher Scientific). Peptides were enriched with μ-Precolumn (0.3 mm i.d. × 5 mm, 5 μm, Thermo Fisher Scientific) and separated on AURORA column (0.075 mm i.d. × 250 mm, 1.6 μm, Ion Opticks Pty Ltd.) using a two-step gradient: 2% to 40% acetonitrile for 110 minutes, followed by 40% to 95% acetonitrile for 5 minutes in the presence of 0.1% formic acid. The compensation voltages for gas-phase fractionation by FAIMS Pro (Thermo Fisher Scientific) were set at −80, −60, and −40 V. The analytic parameters of Orbitrap Fusion Lumos were as follows: resolution of full scans, 50,000; scan range (m/z), 350 to 1,500; maximum injection time of full scans, 50 milliseconds; AGC target of full scans, 4 × 10^5^; dynamic exclusion duration, 30 seconds; cycle time of data-dependent MS/MS acquisition, 2 seconds; activation type, HCD; detector of MS/MS, ion trap; maximum injection time of MS/MS, 35 milliseconds; and AGC target of MS/MS, 1 × 10^4^. The MS/MS spectra were searched against the *Homo sapiens* protein sequence database in SwissProt using Proteome Discoverer 3.1 software (Thermo Fisher Scientific, RRID: SCR_014477), in which peptide identification filters were set at “false discovery rate <1%.” Label-free relative quantification analysis for proteins was performed with the default parameters of the Minora Feature Detector, Feature Mapper, and Precursor Ions Quantifier nodes in Proteome Discoverer 3.1 software. Gene Ontology (GO) analysis was performed using Metascape (RRID: SCR_016620) to identify pathways more enriched by ZSTK474 than by alpelisib. Phosphopeptides with greater than twofold changes in abundance were subjected to GO analysis. To identify kinases with greater changes in activity upon treatment with ZSTK474 than with alpelisib, KSEA was performed (https://casecpb.shinyapps.io/ksea/).

### CRISPR/Cas9-mediated knockout

To generate *PIK3CB*- or *PIK3CD*-knockout cells, Cas9 was first stably expressed in cells using a lentivirus vector (lentiCas9-Blast, Addgene). CRISPR RNA (crRNA) targeting *PIK3CB* (5′-GUG​AUU​GUG​GGA​AAA​UCU​CUguuu​uag​agc​uau​gcu-3′) and trans-activating CRISPR RNA (tracrRNA; 5′-AAA​CAG​CAU​AGC​AAG​UUA​AAA​UAA​GGC​UAG​UCC​GUU​AUC​AAC​UUG​AAA​AAG​UGG​CAC​CGA​GUC​GGU​GCU-3′) were purchased from Integrated DNA Technologies. crRNA:tracrRNA duplexes were prepared by mixing individual crRNAs in a 1:1 mol/L ratio with tracrRNA, followed by heating at 95°C for 5 minutes and annealing at room temperature for 5 to 10 minutes. gRNA vectors for *PIK3CD* were purchased from VectorBuilder, Inc. The target sequence for *PIK3CD* was 5′-TCT​TCA​CGC​GGT​CGC​CCT​CA-3′ or 5′-AGA​GCG​GCTCAT​ACT​GGG​CG-3′. Cas9-expressing cells were plated in six-well plates and transfected with 22 nmol/L crRNA:tracrRNA or 1.3 μg/mL gRNA vector using Lipofectamine 2000 (Thermo Fisher Scientific) according to the manufacturer’s instructions. Single colonies of the transfected cells were obtained using the limited dilution method and expanded. Knockout of *PIK3CB* and/or *PIK3CD* was verified by measuring protein expression using immunoblotting and analyzing the sequence of the genomic DNA region targeted by the gRNAs using Sanger sequencing.

### Mouse xenograft model

Aska-SS or SYO-1 (RRID: CVCL_7146) cells were subcutaneously inoculated into the backs of female BALB/c nude mice (The Jackson Laboratory). When tumors reached 100 to 300 mm^3^ in volume, the mice were randomized into the vehicle control, alpelisib, AZD6482, idelalisib, or combination treatment group. Compounds were dissolved in solvent [10% DMSO + 30% PEG400 (FUJIFILM Wako Pure Chemicals) + 1% Tween-80 (Sigma-Aldrich) + 59% water]. Alpelisib (10 or 20 mg/kg) and idelalisib (30 mg/kg) were administered orally, whereas AZD6482 (20 mg/kg) was intraperitoneally injected. For all drugs, treatment was performed once a day for 15 days in the SYO-1 xenograft model and for 21 days in the Aska-SS model, with treatment omitted on days 5, 6, 12, 13, 19, and 20 after the first day of administration (day 1). The tumor volume (TV) was monitored twice a week by measuring the length (L) and width (W) of the subcutaneous tumor mass using calipers with the following formula: TV = (L × W^2^)/2. For immunoblotting, tumor samples were resected from mice 4 hours after drug administration on day 15 in the SYO-1 xenograft model or on day 21 in the Aska-SS xenograft model. The experimental protocol was approved by the Institutional Animal Experimental Committee at Japanese Foundation for Cancer Research.

### Statistical analysis

The significance of differences between groups was determined with Excel (Microsoft) and EZR software using one-way repeated-measures ANOVA with Bonferroni *post hoc* test, one-way ANOVA with Dunnett *post hoc* test, or the Kruskal–Wallis test with Steel *post hoc* test. *P* < 0.05 was considered statistically significant.

## Results

### Characterization of the mutation status and the mRNA/protein expression of class I PI3K isoforms and PI3K-related genes in sarcoma cell lines

We first investigated the mutation status of class I PI3K isoforms (*PIK3CA*, *PIK3CB*, *PIK3CD*, and *PIK3CG*), *PTEN*, *KRAS*, and RTKs in a sarcoma cell line panel comprising 23 cell lines, including 11 TRS cell lines [six SS, two Ewing sarcoma, one ARMS, and two alveolar soft part sarcoma (ASPS) cell lines], by whole-exome sequencing. We found two reported gain-of-function (GOF) hotspot mutations, including *PIK3CA* H1047R in Yamato-SS and *PIK3CD* E1021K in SW684; however, such GOF mutations in PI3K isoforms seemed to be uncommon in both TRS and non-TRS cells. By contrast, we found the *BRAF* V600E mutation in 4 of 23 cell lines (A-673 TRS cells and GCT, SW872, and SW982 non-TRS cells), the *KRAS* G12C mutation in SW1353 cells, and the *NRAS* Q61K and Q61H mutations in HT-1080 and RD cells (all from non-TRS), suggesting that activation of the RAS/RAF pathway is more common in sarcoma cells (Table 1; Supplementary Table S2). We further investigated alterations in these genes in clinical samples of SS, Ewing sarcoma, ARMS, and breast cancer using the cBioPortal database. We found that mutations or amplifications of *PIK3CA* and *ERBB2* were present in approximately 40% and 11% of breast cancers, respectively, whereas in TRSs, alteration frequencies of all examined PI3K isoforms and PI3K-related genes were below 5% in clinical samples (Supplementary Fig. S1).

**Table 1.** Fusion genes and mutations of PI3K-related genes in sarcoma cell lines.

Subtype	Cell line	Fusion gene	*PIK3CA*	*PIK3CB*	*PIK3CD*	*PIK3CG*	*PTEN*	*BRAF*	*KRAS*	*NRAS*	*HRAS*
SS (with fusion)	Aska-SS	*SS18::SSX1*	​	​	​	S442Y	​	​	​	​	​
	SYO-1	*SS18::SSX2*	​	​	​	​	​	​	​	​	​
	Yamato-SS	*SS18::SSX1*	H1047R	​	​	​	​	​	​	​	​
	Fuji	*SS18::SSX2*	​	​	​	​	​	​	​	​	​
	HS-SY-II	*SS18::SSX1*	​	​	​	​	​	​	​	​	​
	1273/99	*SS18::SSX2*	​	​	​	N522S	​	​	​	​	​
Ewing sarcoma	A-673	*EWSR1::FLI1*	​	​	​	S442Y	​	V600E	​	​	​
	RD-ES	*EWSR1::FLI1*	​	​	​	​	​	​	​	​	​
ARMS	SJCRH30	*PAX3::FOXO1*	​	​	​	​	​	​	​	​	​
ASPS	ASPS-1	*ASPSCR1::TFE3*	​	​	​	​	​	​	​	​	​
	ASPS-KY	*ASPSCR1::TFE3*	​	​	​	​	​	​	​	​	​
Fibrosarcoma	HT-1080	—	​	​	​	​	​	​	​	Q61K	​
	SW684	—	​	​	E1021K	​	​	​	​	​	​
Giant cell sarcoma	GCT	—	​	​	​	​	P204L	V600E	​	​	​
Liposarcoma	SW872	—	​	​	​	S442Y	​	V600E	​	​	​
SS (without fusion)	SW982	—	​	​	​	​	​	V600E	​	​	​
Chondrosarcoma	SW1353	—	I391M	​	​	​	​	​	G12C	​	​
Uterine sarcoma	MES-SA	—	​	​	​	​	​	​	​	​	​
Leiomyosarcoma	SK-UT-1	—	R88Q	​	​	​	​	​	​	​	R73C
Embryonic rhabdomyosarcoma	RD	—	​	​	​	​	​	​	​	Q61H	​
Osteosarcoma	Saos-2	—	​	​	​	S442Y	​	​	​	​	​
	HOS	—	I391M	​	​	S442Y	​	​	​	​	​
	KHOS-240S	—	I391M	​	​	S442Y	​	​	​	​	​

**Figure 1.**
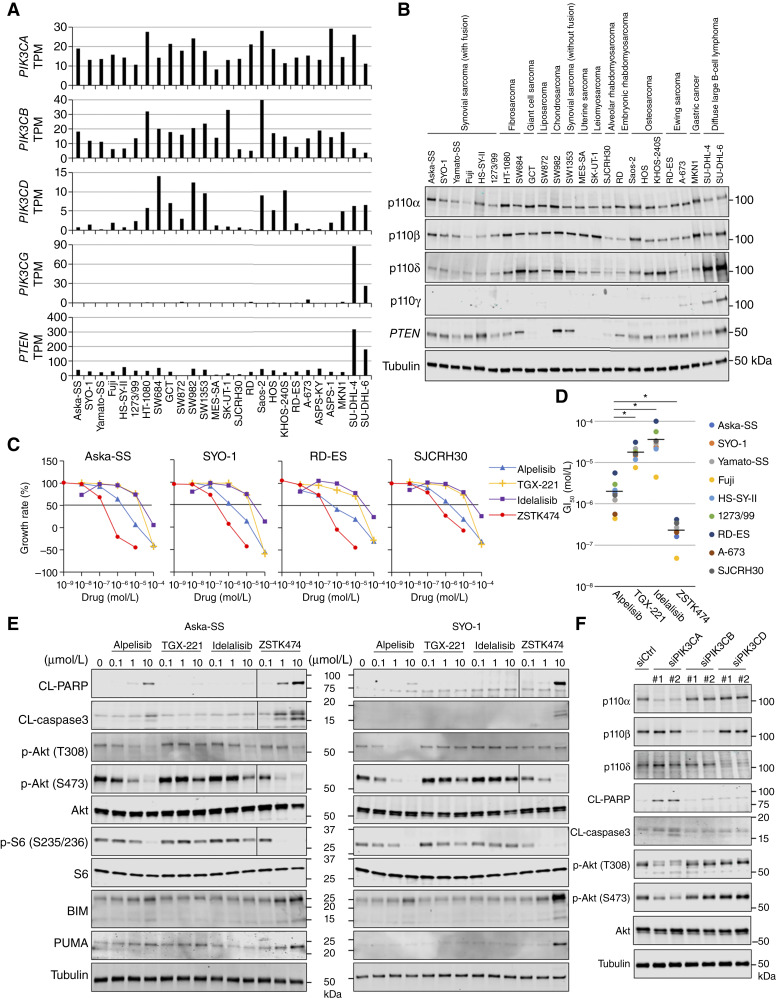
Among class I PI3K isoforms, PI3Kα is the dominant isoform in TRSs. **A,** The expression of *PIK3CA*, *PIK3CB*, *PIK3CD*, *PIK3CG*, and *PTEN* in the indicated sarcoma cell lines, a gastric cancer cell line, and diffuse large B-cell lymphoma cell lines determined by RNA-seq. **B,** Immunoblots presenting the expression of p110α, p110β, p110δ, p110γ, and *PTEN* in the indicated cell lines. Tubulin was used as a loading control. **C,** Concentration–response curves of alpelisib, TGX-221, idelalisib, and ZSTK474 in the indicated TRS cell lines. **D,** Dot plots presenting differences in the GI_50_ values of alpelisib, TGX-221, idelalisib, and ZSTK474 in the indicated TRS cell lines. GI_50_ value was calculated using the growth curves presented in **C** and Supplementary Fig. S5. Statistical analysis was performed by one-way repeated-measures ANOVA with Bonferroni *post hoc* test. *, *P* < 0.05. **E,** Immunoblots of the indicated proteins in Aska-SS and SYO-1 cells treated with alpelisib, TGX-221, idelalisib, and ZSTK474 for 48 hours. Tubulin was used as a loading control. **F,** Immunoblots of the indicated proteins in Aska-SS cells transfected with siRNAs specific for *PIK3CA*, *PIK3CB*, and *PIK3CD*. Tubulin was used as a loading control.

We next examined the mRNA and protein expression of class I PI3K isoforms, *PTEN*, and RTKs in sarcoma cell lines by RNA-seq and immunoblotting, respectively. A previous study reported that PI3Kα and PI3Kβ are ubiquitously expressed, whereas PI3Kδ and PI3Kγ are selectively expressed in leukocytes (9). Therefore, we used the MKN1 gastric carcinoma cell line (19) and the SU-DHL-4 and SU-DHL-6 DLBCL cell lines (20) as positive controls for PI3Kα/β and PI3Kδ/γ, respectively. Among the cell lines examined, mRNA and protein levels were highly correlated for each class I PI3K isoform and *PTEN*, with higher mRNA levels corresponding to higher protein levels (Supplementary Fig. S2). Whereas PI3Kα (p110α) and PI3Kβ (p110β) expression was detected at both the mRNA and protein levels in all sarcoma cell lines examined including TRS cells at levels comparable with those in MKN1 cells, PI3Kδ (p110δ) was unexpectedly detected in most cell lines at slightly weaker levels compared with that in lymphoma cell lines. Conversely, PI3Kγ (p110γ) expression was barely detected in all sarcoma cell lines (Fig. 1A and B). Regarding PTEN, most sarcoma cell lines including TRS cells displayed substantial PTEN protein expression; however, GCT, SW872, MES-SA, and SK-UT-1 did not express this protein (Fig. 1B). Concerning RTKs, *EGFR*, *ERBB2*, *INSR*, *IGF1R*, and *MET* displayed broad mRNA expression across the examined sarcoma cell lines, whereas *ERBB3*, *ERBB4*, *KIT*, and *VEGFR* were expressed in particular sarcoma cell lines, i.e., *ERBB3* in SJCRH30 and RD cells; *ERBB4* in A-673 cells; *KIT* in Yamato-SS, Saos-2, and RD-ES cells; and *VEGFR* in ASPS-1 cells. In particular, *PDGFRA* displayed SS subtype-specific expression, and it was expressed in all six SS cell lines (Supplementary Fig. S3A). To examine whether similar expression patterns are observed in clinical specimens, we analyzed the expression of these genes in clinical datasets for SS, Ewing sarcoma, breast cancer, and DLBCL using RNA-seq data from the GEO database. Consistent with the cell line data, *PIK3CA* and *PIK3CB* were expressed in SS and Ewing sarcoma at levels comparable with those observed in breast cancer, a carcinoma. *PIK3CD* expression was detectable in SS and Ewing sarcoma, although at lower levels than in DLBCL. *PIK3CG* expression was observed in Ewing sarcoma but was minimal in SS. Among RTKs, *EGFR* and *PDGFRA* tended to show higher expression in SS, *ERBB4* and *KIT* in Ewing sarcoma, and *ERBB2* and *ERBB3* in breast cancer (Supplementary Fig. S3B).

### Inhibition of PI3Kα, but not PI3Kβ or PI3Kδ, impairs PI3K signaling, survival, and growth in TRS cells

We next investigated the role of the three class IA PI3K isoforms (PI3Kα, PI3Kβ, and PI3Kδ) commonly expressed in TRS cell lines. To this end, we used PI3K isoform–specific inhibitors including alpelisib for PI3Kα, TGX-221 for PI3Kβ, and idelalisib for PI3Kδ, as well as the pan-class I PI3K inhibitor ZSTK474. Based on published data by our group and others, these inhibitors exhibited specific and comparable kinase-inhibitory activity against their respective target isoforms at the biochemical level (Supplementary Fig. S4A; refs. 21–24). Because PI3Kα, PI3Kβ, and PI3Kδ are the primary isoforms contributing to the activation of PI3K signaling and cell growth in *PIK3CA*-mutated cancers, *PTEN*-deficient cancers, and B-cell lymphomas, respectively (25–30), we used MKN1 gastric cancer cells with a *PIK3CA* hotspot mutation (E545K; ref. 19), PC-3 prostate cancer cells with *PTEN* loss (19), and SU-DHL-4 B-cell lymphoma cells (20) as positive controls to confirm whether each isoform-selective inhibitor selectively targets its corresponding isoform and cancer cell type at the cellular level. Expectedly, among the isoform-specific inhibitors, alpelisib most effectively decreased the phosphorylation of PI3K signaling molecules including Akt and S6 in MKN-1 cells, whereas TGX-221 and idelalisib were most effective in PC-3 and SU-DHL-4 cells, respectively, with effective concentrations of approximately 1 μmol/L, which were similar across all three inhibitors (Supplementary Fig. S4B). Along with the suppression of PI3K signaling, alpelisib, TGX-221, and idelalisib exhibited the strongest antiproliferative effects on MKN1, PC-3, and SU-DHL-4, respectively (Supplementary Fig. S4C). These results suggest that these PI3K inhibitors selectively inhibit their respective target isoforms over a comparable concentration range at the cellular level, as originally intended.

We then examined the effects of these PI3K inhibitors on TRS cells. Among the isoform-specific inhibitors, alpelisib exhibited the strongest effects on the downregulation of PI3K-downstream signaling and cell growth against TRS cell lines, including SS, Ewing sarcoma, and ARMS cells (Fig. 1C–E; Supplementary Fig. S5). Similar results were obtained with specific siRNAs targeting the catalytic subunit of each class IA PI3K isoform; i.e., treatment with specific siRNAs for *PIK3CA* decreased Akt phosphorylation, whereas siRNAs for *PIK3CB* or *PIK3CD* failed to decrease this phosphorylation (Fig. 1F). In parallel to the suppression of PI3K signaling, the inhibition of PI3Kα by alpelisib or siRNAs increased the cleavage of PARP and caspase-3, indicating the induction of apoptosis, whereas inhibition of PI3Kβ and PI3Kδ had no such effects (Fig. 1E and F). These results suggest that among class IA PI3K isoforms, PI3Kα primarily contributes to the transduction of PI3K signaling and survival in TRS cells.

### Simultaneous inhibition of PI3Kα with PI3Kβ and/or PI3Kδ synergistically induces apoptosis and impairs proliferation and cell-cycle progression in TRS cells

**Figure 2.**
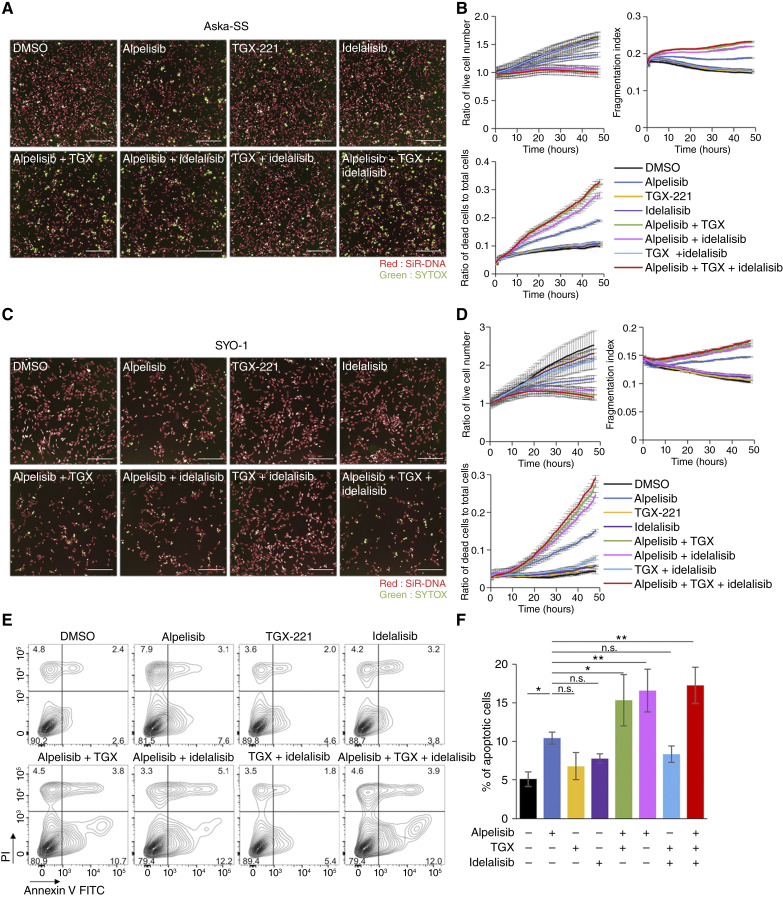
Simultaneous inhibition of PI3Kα with PI3Kβ and/or PI3Kδ results in synergistic antitumor effects and apoptosis induction in TRS cells. **A–D,** Fluorescence time-lapse imaging analysis in Aska-SS (**A** and **B**) and SYO-1 cells (**C** and **D**) stained with SiR-DNA and SYTOX Green followed by treatment with alpelisib, TGX-221, and idelalisib alone or in combination for 48 hours. Drugs were added at 1 µmol/L for Aska-SS cells and at 8 μmol/L for SYO-1 cells. Representative images of Aska-SS (**A**) and SYO-1 cells (**C**) at 48 hours are presented. The growth rate of live cells (top left), the fragmentation index (top right), and the proportion of dead cells to total cells (bottom) in Aska-SS (**B**) and SYO-1 cells (**D**) were calculated by quantitative image analysis. Data are presented as the mean ± SD (*n* = 5). **E,** Representative flow cytometry plots of Aska-SS cells stained with Annexin V–FITC/PI after treatment with alpelisib, TGX-221, and idelalisib alone or in combination for 48 hours. **F,** Summary of Annexin V–FITC-positive cells (%) in **E**. Data are presented as the mean ± SD (*n* = 3). Statistical analysis was performed by one-way ANOVA with Dunnett *post hoc* test. n.s., not significant; *, *P* < 0.05; **, *P* < 0.01.

Comparing the antitumor effects of isoform-selective inhibitors and the pan-class I PI3K inhibitor ZSTK474 in Fig. 1C–E and Supplementary Fig. S5, treatment with ZSTK474 resulted in significantly stronger antitumor effects, including downregulation of PI3K downstream signals and induction of apoptosis associated with the upregulation of BIM and PUMA (15) in the SS cell lines Aska-SS and SYO-1. These data prompted us to investigate whether the simultaneous inhibition of multiple class IA PI3K isoforms enhances antitumor effects and apoptosis induction in TRS cells compared with the inhibition of a single PI3K isoform. To address this possibility, we used time-lapse imaging to monitor the temporal changes in live and dead cells following treatment with either a single or multiple isoform-specific inhibitors. To this end, nuclei were stained with the low-toxic DNA dye SiR-DNA, whereas dead cells were identified by nuclear staining with SYTOX Green, which indicates membrane permeability associated with loss of cell viability. Inhibition of PI3Kα alone by alpelisib slightly decreased the number of live cells and increased the number of dead cells in the Aska-SS, SYO-1, and SJCRH30 cell lines compared with the effects of dimethyl sulfoxide (DMSO), whereas single inhibition of PI3Kβ and PI3Kδ by TGX-221 and idelalisib, respectively, did not exert such an effect (Fig. 2A–D; Supplementary Fig. S6A and S6B). By contrast, when alpelisib was combined with TGX-221 and/or idelalisib, the number of live cells significantly decreased compared with alpelisib treatment alone, and the number of dead cells markedly increased. Conversely, the combination with TGX-221 and idelalisib had no such effects (Fig. 2A–D; Supplementary Fig. S6A and S6B). To assess the progression of apoptosis, we calculated the fragmentation index, the CV of SiR-DNA intensity within the region of interest of each nucleus. Healthy nuclei have low CVs, whereas fragmented nuclei undergoing apoptosis exhibit high CVs (31). Along with the increase in the number of SYTOX-positive cells, the fragmentation index was significantly increased by treatment with alpelisib alone but not by TGX-221 or idelalisib alone. However, the combination of TGX-221 and/or idelalisib with alpelisib greatly increased the fragmentation index compared with that of alpelisib alone (Fig. 2A–D; Supplementary Fig. S6A and S6B). Annexin V/PI staining also demonstrated that treatment with alpelisib increased the number of apoptotic cells, and the combination of TGX-221 and/or idelalisib with alpelisib further enhanced apoptosis induction compared with the effect of alpelisib alone (Fig. 2E and F). We next investigated the combination effect of isoform-selective inhibitors on MKN1 cells harboring the *PIK3CA* E545K mutation. As previously mentioned, alpelisib exerted comparably strong antitumor effects on MKN1 cells as ZSTK474 (Supplementary Fig. S4B and S4C). In addition, time-lapse imaging clearly revealed that alpelisib alone exerted a strong anticancer effect, but the combination of TGX-221 and/or idelalisib with alpelisib failed to enhance the effect of alpelisib (Supplementary Fig. S6C and S6D).

**Figure 3.**
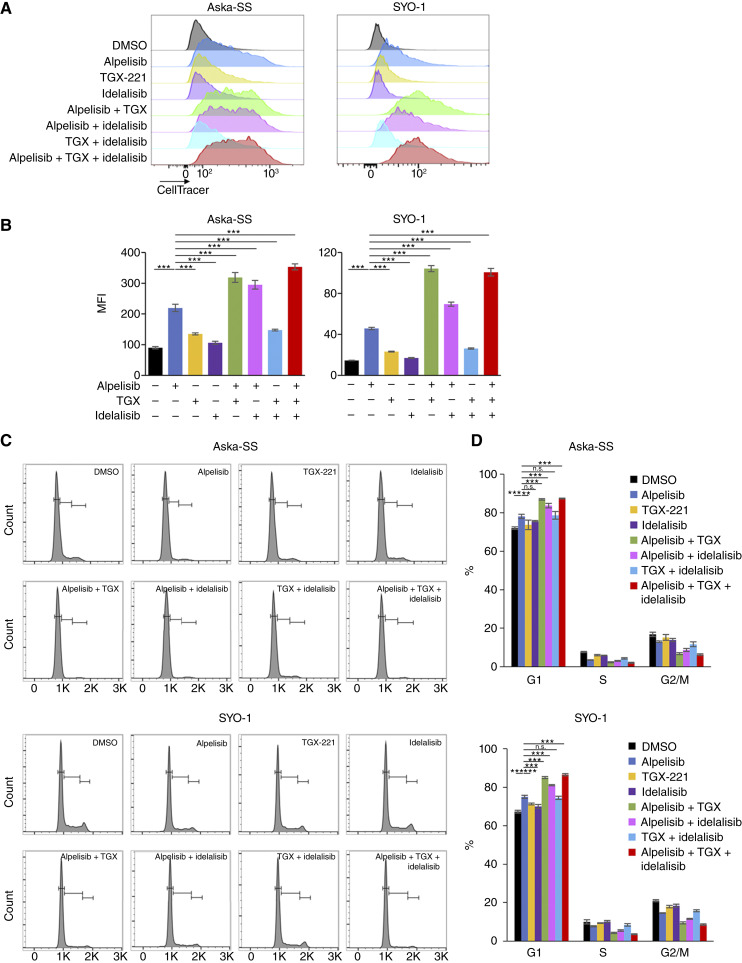
Simultaneous inhibition of PI3Kα with PI3Kβ and/or PI3Kδ impairs proliferation and cell-cycle progression in TRS cells. **A,** Representative flow cytometry histograms assessing the proliferation of Aska-SS or SYO-1 cells stained with CellTracer Yellow after treatment with alpelisib, TGX-221, and idelalisib alone or in combination. Drugs were added at 4 μmol/L for Aska-SS cells and at 8 μmol/L for SYO-1 cells. Proliferation was examined by CellTracer Yellow dilution after culture with or without the drugs for 6 days. **B,** Summaries of the median fluorescence intensity (MFI) of CellTracer Yellow in the cell proliferation assay presented in **A**. Data are presented as the mean ± SD (*n* = 4). Statistical analysis was performed by one-way ANOVA with Dunnett *post hoc* test. ***, *P* < 0.001. **C,** Representative flow cytometry histograms of the cell-cycle distribution (G1, S, and G2/M) in Aska-SS (top) and SYO-1 cells (bottom) treated with alpelisib, TGX-221, and idelalisib alone or in combination for 48 hours. Drugs were added at 4 μmol/L for Aska-SS cells and at 8 μmol/L for SYO-1 cells. **D,** Summaries of the cell-cycle distribution (G1, S, and G2/M) presented in **C**. Data are presented as the mean ± SD (*n* = 4). Statistical analysis was performed by one-way ANOVA with Dunnett *post hoc* test. n.s, not significant; **, *P* < 0.01; ***, *P* < 0.001.

We next examined the effect of isoform-selective PI3K inhibitors, both alone and in combination, on cell proliferation and cell-cycle progression in TRS cells. To assess cell proliferation, we used a fluorescent dye that pulse-stains cells to track their proliferation. Proliferation was measured by dye dilution, with the fluorescence signal halving with each division. The stained cells were incubated with or without isoform-selective inhibitors for 6 days, and the remaining fluorescence intensity within the cells was measured by flow cytometry. Treatment with alpelisib resulted in the significant accumulation of cells with higher fluorescence intensity compared with the vehicle control, TGX-221 alone, or idelalisib alone in Aska-SS, SYO-1 and SJCRH30 cells (Fig. 3A and B; Supplementary Fig. S7A and S7B). This suggests that PI3Kα primarily contributes to the proliferation of TRS cells. As expected, the combination of TGX-221 and/or idelalisib with alpelisib resulted in significantly higher fluorescence intensity compared with that of alpelisib alone (Fig. 3A and B; Supplementary Fig. S7A and S7B). Conversely, MKN1 cells treated with alpelisib alone, but not with TGX-221 and idelalisib alone, exhibited higher fluorescence intensity, and the addition of TGX-221 and/or idelalisib failed to enhance the effect of alpelisib (Supplementary Fig. S7A and S7B). Along with the inhibition of proliferation, treatment with alpelisib significantly increased G1 phase accumulation in Aska-SS, SYO-1, SJCRH30, and MKN1 cells, whereas TGX-221 or idelalisib alone had little effect on G1 phase accumulation in TRS cells (Fig. 3C and D; Supplementary Fig. S7C and S7D). Moreover, the combination of TGX-221 and/or idelalisib with alpelisib increased the G1 phase population compared with the effect of alpelisib alone in TRS cells, but not in MKN1 cells (Fig. 3C and D; Supplementary Fig. S7C and S7D). These results indicate that PI3Kα has the primary role in promoting proliferation, cell-cycle progression, and cell survival in TRS cells including Aska-SS, SYO-1, and SJCRH30 cells, whereas PI3Kβ and PI3Kδ can compensate for these functions when PI3Kα is unavailable.

### Simultaneous inhibition of PI3Kα with PI3Kβ and/or PI3Kδ enhances growth inhibition but does not induce apoptosis in diverse nonsarcoma cancer cell lines

**Figure 4.**
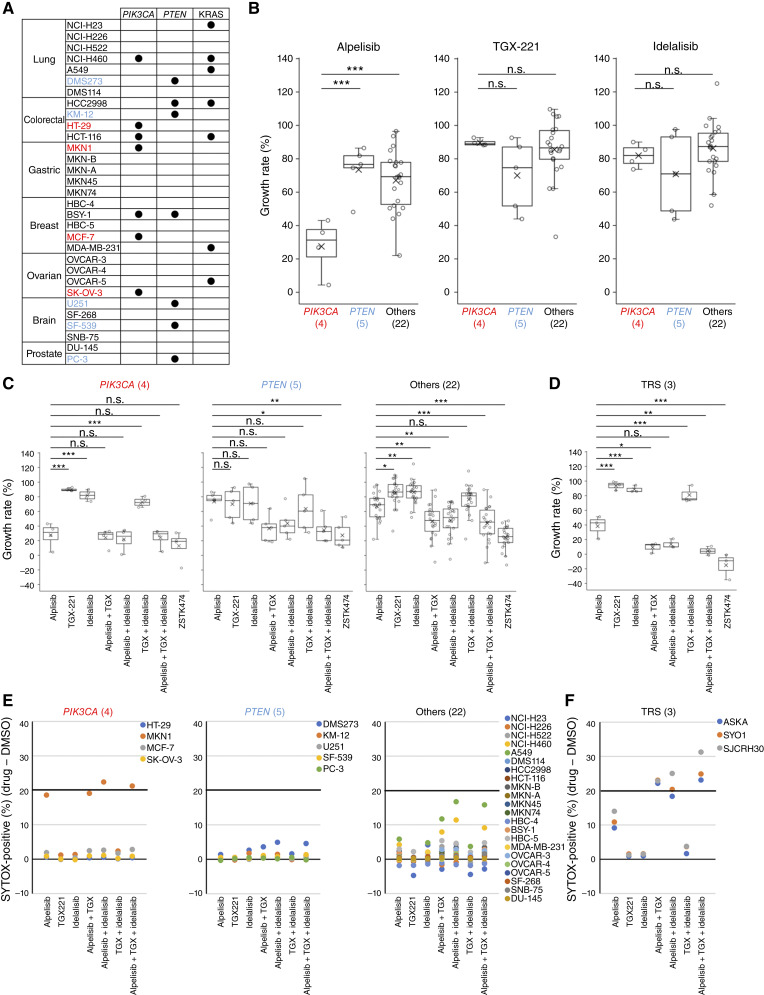
Simultaneous inhibition of PI3Kα with PI3Kβ and/or PI3Kδ enhances growth inhibition but does not induce apoptosis in diverse nonsarcoma cancer cell lines. **A,** The list of cell lines used in this study and their mutation status for *PIK3CA*, *PTEN*, and *KRAS*. Cell lines shown in red are classified as the *PIK3CA*-mutated group (*PTEN*-wild and *KRAS*-wild), and those in blue are classified as the *PTEN*-deficient group (*PIK3CA*-wild and *KRAS*-wild). **B,** Box plots showing growth rate (%) of nonsarcoma cells treated with indicated drugs at 8 μmol/L for 48 hours, comparing between *PIK3CA*-mutated, *PTEN*-deficient, and other cell lines. Statistical analysis was performed by one-way ANOVA with Dunnett *post hoc* test. n.s., not significant; ***, *P* < 0.001. **C** and **D,** Box plots showing growth rate (%) of nonsarcoma cells (**C**) and TRS cells (**D**) at 48 hours, comparing the indicated drug treatments. Statistical analysis was performed by one-way ANOVA with Dunnett *post hoc* test. n.s., not significant; *, *P* < 0.05; **, *P* < 0.01; ***, *P* < 0.001. **E** and **F,** Dot plots showing SYTOX positivity (%) of nonsarcoma cells (**E**) and TRS cells (**F**) at 48 hours, comparing the indicated drug treatments.

We next tested whether the cooperative roles of class IA PI3K isoforms in the proliferation and survival of TRS cells were observed across diverse cancer cell types. To this end, our JFCR39 cell line panel (19), consisting of 39 cancer cell lines derived from nine different tissues, was used. Cell lines were classified into three groups: *PIK3CA*-mutated (*PTEN*-wild and *KRAS*-wild, four cell lines), *PTEN*-deficient (*PIK3CA*-wild and *KRAS*-wild, five cell lines), and others (22 cell lines; Fig. 4A). Eight cell lines were not tested. The percentages of live and dead cells following treatment with either a single or multiple isoform-specific inhibitors were measured by time-lapse imaging. Upon treatment with a single agent, alpelisib significantly inhibited the growth of *PIK3CA*-mutated cell lines compared with *PTEN*-deficient and the other cell lines. TGX-221 and idelalisib showed only weak effects in most cell lines tested, although they were slightly more effective in *PTEN*-deficient cell lines than the other groups (Fig. 4B; Supplementary Fig. S8A). In the comparison between single-agent and combination treatments, *PIK3CA*-mutated cell lines such as MKN1 were highly sensitive to alpelisib among single-agent treatments, whereas no enhanced effect was observed with inhibition of multiple PI3K isoforms by either combination treatment or the pan-PI3K inhibitor ZSTK474. In *PTEN*-deficient cell lines, although all isoform-specific inhibitors alone showed only modest efficacy, the combination of TGX-221 and/or idelalisib with alpelisib significantly enhanced the single-agent effect. In other nonsarcoma cell lines, although alpelisib showed the highest efficacy among isoform-specific inhibitors alone, the combination and ZSTK474 treatment showed even higher efficacy than any single agent (Fig. 4C; Supplementary Fig. S8B). Similarly, TRS cell lines exhibited high sensitivity to alpelisib, and enhanced effects were observed with the simultaneous inhibition of PI3Kα with PI3Kβ and/or PI3Kδ (Fig. 4D). These results indicate that in *PIK3CA*-mutated cells, PI3Kα primarily functions, whereas in the other nonsarcoma cancer cells, including *PTEN*-deficient cells, PI3Kα with PI3Kβ and/or PI3Kδ cooperatively regulate cell proliferation, similar to TRS cells.

As for cell survival, few cancer cell lines tested, with the exception of MKN1, showed significant cell death exceeding 20% SYTOX-positive cells (Fig. 4E; Supplementary Fig. S8C). MKN1 cells exhibited cell death upon PI3Kα inhibition, but no enhancement was observed with simultaneous inhibition of multiple PI3K isoforms. By contrast, in TRS cells, PI3Kα inhibition induced only modest cell death, but the effect was markedly enhanced when PI3Kα with PI3Kβ and/or PI3Kδ were simultaneously inhibited (Fig. 4F). These results suggest that PI3K signaling regulates survival only in TRS cells and some *PIK3CA*-mutated cells. Given that PI3Kα is the responsible isoform in *PIK3CA*-mutated cells, the cooperative regulation among class IA PI3K isoforms in cell survival is observed especially in TRS cells.

### Phosphoproteomic analysis reveals stronger inhibition of PI3K-downstream Akt/mTOR signaling by ZSTK474 than by alpelisib

**Figure 5.**
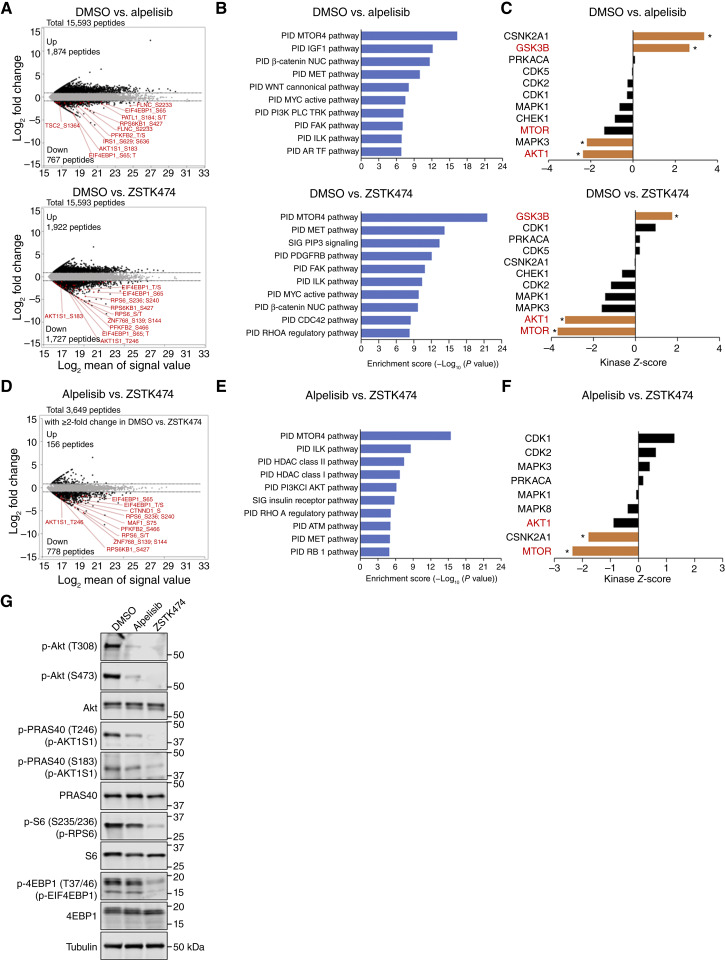
Phosphoproteomic analysis reveals that ZSTK474, a pan-PI3K inhibitor, more potently inhibits Akt/mTOR signaling than the PI3Kα-specific inhibitor alpelisib. **A,** MA plots of phosphopeptides in DMSO-treated Aska-SS cells vs. alpelisib-treated Aska-SS cells (top) or DMSO-treated Aska-SS cells vs. ZSTK474-treated Aska-SS cells (bottom). Drugs were added at 1 μmol/L for 1 hour. The dotted lines indicate the boundary of the twofold change. Phosphopeptides in Akt/mTOR signaling are highlighted by red plots. Among them, representative phosphopeptides in Akt/mTOR signaling are indicated. **B,** GO analysis of phosphoproteins with greater than twofold changes in abundance between DMSO and alpelisib treatment (top) or between DMSO and ZSTK474 treatment (bottom). **C,** KSEA revealing activated or inactivated kinases in DMSO-treated Aska-SS cells vs. alpelisib-treated Aska-SS cells (top) or DMSO-treated Aska-SS cells vs. ZSTK474-treated Aska-SS cells (bottom). Positive kinase *Z*-scores indicate that the kinase is activated in Aska-SS cells treated with alpelisib or ZSTK474 compared with the findings in Aska-SS cells treated with DMSO, whereas negative kinase *Z*-scores indicate that the kinase is inactivated in Aska-SS cells treated with alpelisib or ZSTK474. *P* values were calculated using the KSEA algorithm. *, *P* < 0.05. **D,** MA plot of phosphopeptides with greater than twofold changes in signals between DMSO and ZSTK474 treatment in alpelisib-treated Aska-SS cells vs. ZSTK474-treated Aska-SS cells. The dotted lines indicate the boundary of the twofold change. Phosphopeptides in Akt/mTOR signaling are highlighted in red plots. Among them, representative phosphopeptides in Akt/mTOR signaling are indicated. **E,** GO analysis of phosphoproteins with greater than twofold changes in abundance between alpelisib and ZSTK474 treatment among phosphoproteins with greater than twofold changes in abundance between DMSO and ZSTK474 treatment. **F,** KSEA revealing activated or inactivated kinases in alpelisib-treated Aska-SS cells vs. ZSTK474-treated Aska-SS cells. *P* values were calculated using the KSEA algorithm. *, *P* < 0.05. **G,** Immunoblots of the indicated proteins in Aska-SS cells treated with alpelisib or ZSTK474 at 1 μmol/L for 1 hour. Tubulin was used as a loading control.

We investigated the mechanism of synergistic cell growth inhibition and apoptosis induction by the simultaneous inhibition of PI3Kα with PI3Kβ and/or PI3Kδ in an unbiased manner. To this end, we performed global phosphoproteomic analysis of Aska-SS cells treated with 1 μmol/L alpelisib, 1 μmol/L ZSTK474, or the vehicle control for 1 hour. Across the three samples, 15,593 phosphopeptides derived from 4,246 unique proteins were identified. Upon treatment with alpelisib or ZSTK474, the abundance of 1,874 and 1,922 phosphopeptides, respectively, was increased by >2-fold, whereas the abundance of 767 and 1,727 phosphopeptides, respectively, was decreased by >2-fold (Fig. 5A). Pathway enrichment analysis revealed that treatment with alpelisib affected the mTOR and IGF1R pathways, and treatment with ZSTK474 affected the mTOR, MET, and PIP3 pathways (Fig. 5B). Concordantly, kinase–substrate enrichment analysis (KSEA) revealed that treatment with alpelisib or ZSTK474 reduced the phosphorylation of AKT1 and mTOR substrates (Fig. 5A and C). To explore the pathways most strongly affected by ZSTK474 treatment compared with alpelisib, we identified 934 phosphopeptides altered by >2-fold by ZSTK474 compared with the findings for alpelisib among 3,649 phosphopeptides altered by >2-fold with ZSTK474 versus untreated cells (Fig. 5D). Pathway enrichment analysis revealed that treatment with ZSTK474 had a greater effect on the mTOR pathway than alpelisib (Fig. 5E). In support of this finding, KSEA revealed that treatment with ZSTK474 more strongly reduced the phosphorylation of mTOR and Akt1 substrates than alpelisib (Fig. 5D and F). Consistently, immunoblotting illustrated that treatment with alpelisib decreased the phosphorylation of Akt (T308 and S473), PRAS40 (T246 and T183), S6 (S235/236), and 4EBP1 (T37/46), whereas treatment with ZSTK474 led to an even greater decrease in the phosphorylation of these PI3K/mTOR downstream molecules (Fig. 5G). These results indicate that the pan-PI3K inhibitor ZSTK474 inhibits Akt/mTOR signaling more strongly than the PI3Kα-specific inhibitor alpelisib, likely because alpelisib allows residual PI3Kβ and PI3Kδ activity whereas ZSTK474 completely inhibits PI3K activity.

### Class IA PI3K isoforms cooperatively regulate Akt/mTOR signaling and thereby cell survival and tumor growth in TRSs

**Figure 6.**
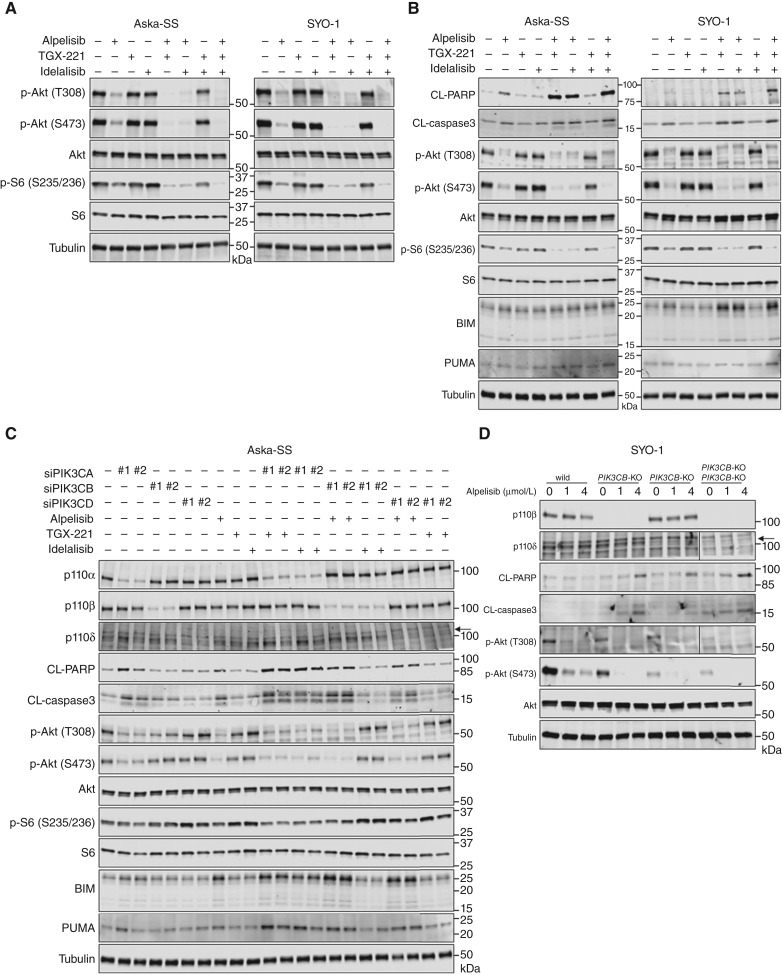
Simultaneous inhibition of PI3Kα with PI3Kβ and/or PI3Kδ significantly suppresses Akt/mTOR signaling. **A** and **B,** Immunoblots of the indicated proteins in Aska-SS and SYO-1 cells treated with alpelisib, TGX-221, and idelalisib alone or in combination at 1 μmol/L for 3 hours (**A**) or 48 hours (**B**). Tubulin was used as a loading control. **C,** Immunoblots of the indicated proteins in Aska-SS cells treated with alpelisib, TGX-221, and idelalisib at 1 μmol/L for 48 hours after transfection with siRNAs specific for *PIK3CA*, *PIK3CB*, or *PIK3CD*. Tubulin was used as a loading control. **D,** Immunoblots of the indicated proteins in SYO-1 cells with *PIK3CB* and/or *PIK3CD* knockout that were treated with alpelisib for 48 hours. Tubulin was used as a loading control.

To validate our hypothesis that simultaneous inhibition of all three class IA PI3K isoforms by ZSTK474 more efficiently inhibits Akt/mTOR signaling than inhibition of PI3Kα alone by alpelisib, we assessed the activation status of Akt/mTOR signaling in TRS cells upon simultaneous inhibition of class IA PI3K isoforms using various combinations of inhibitors, siRNAs, and gene knockout via CRISPR/Cas9. Pharmacologic inhibition of PI3Kα with alpelisib decreased Akt phosphorylation at T308 and S473 and S6 phosphorylation at S235/236, whereas inhibition of PI3Kα (alpelisib) simultaneously with PI3Kβ (TGX-221) and/or PI3Kδ (idelalisib) inhibition further decreased the phosphorylation of these PI3K/mTOR downstream factors (Fig. 6A). However, combination treatment with TGX-221 and idelalisib slightly reduced Akt and S6 phosphorylation (Fig. 6A). In accordance with the downregulation of Akt/mTOR signaling, treatment with alpelisib in combination with TGX-221 and/or idelalisib strongly induced PARP and caspase-3 cleavage and induced either BIM or PUMA in Aska-SS and SYO-1 cells, indicating enhanced apoptosis induction compared with the effect of alpelisib alone (Fig. 6B). Similar results were obtained in other TRS cell lines, including RD-Ewing sarcoma and SJCRH30 cells (Supplementary Fig. S9). In addition, combination treatment with PI3K isoform–specific inhibitors and siRNAs produced similar results (Fig. 6C). To further investigate the compensatory role of PI3Kβ and PI3Kδ with PI3Kα in signal activation and cell survival in TRSs, we used the CRISPR/Cas9 system to knock out *PIK3CB* and/or *PIK3CD* in SYO-1 cells. Upon treatment with alpelisib alone, SYO-1 cells lacking *PIK3CB* and/or *PIK3CD* exhibited a significant decrease in Akt phosphorylation, resulting in strong apoptosis induction, as determined by PARP and caspase-3 cleavage, compared with that in parental SYO-1 cells (Fig. 6D). Taken together, among the class IA PI3K isoforms, PI3Kα primarily transduces PI3K signals, thereby contributing to cell survival and proliferation in TRSs, whereas PI3Kβ and PI3Kδ play compensatory roles with PI3Kα. Therefore, inhibition of all three isoform is required to achieve effective antitumor activity in such sarcoma cells.

**Figure 7.**
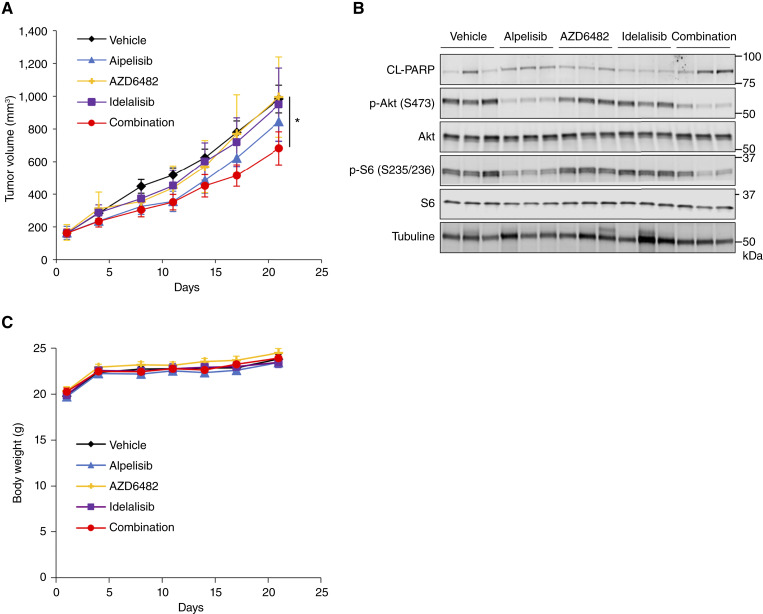
Simultaneous inhibition of PI3Kα, PI3Kβ, and PI3Kδ leads to significant tumor growth inhibition associated with suppression of PI3K signaling and apoptosis induction in the xenograft model. **A,** Growth curves of Aska-SS xenograft tumors in mice administered alpelisib (10 mg/kg), AZD6482 (20 mg/kg), and idelalisib (30 mg/kg) alone or in combination. Data are presented as the mean ± SD (*n* = 6). Statistical analysis was performed by the Kruskal–Wallis test with Steel *post hoc* test. *, *P* < 0.05. **B,** Immunoblots of the indicated proteins in mice bearing Aska-SS xenograft tumors administered alpelisib, AZD6482, and idelalisib alone or in combination. Tubulin was used as a loading control. **C,** Body weight changes of mice bearing Aska-SS xenograft tumors administered alpelisib, AZD6482, and idelalisib alone or in combination.

To confirm the aforementioned *in vitro* findings in an *in vivo* model, we examined whether the simultaneous inhibition of class IA PI3K isoforms would also be effective in xenograft models. As TGX-221 is insoluble in water, AZD6482, its water-soluble analog, was used as a PI3Kβ-specific inhibitor (32). The pharmacokinetic profiles of ZSTK474 and other PI3K isoform inhibitors differ in mice, making it difficult to control for these differences. Therefore, for comparison with PI3K isoform inhibitor monotherapy, we used combination treatment with PI3K isoform inhibitors rather than ZSTK474. The administration of alpelisib slightly inhibited the growth of Aska-SS xenografts, whereas AZD6482 and idelalisib had minimal effects on tumor growth (Fig. 7A). Importantly, combination treatment with alpelisib, AZD6482, and idelalisib significantly inhibited tumor growth in Aska-SS and SYO-1 xenografts (Fig. 7A; Supplementary Fig. S10A). In support of these findings, the administration of alpelisib alone slightly induced apoptosis, whereas the combination of all three drugs greatly enhanced the induction of apoptosis as determined by PARP cleavage in Aska-SS and SYO-1 xenograft models (Fig. 7B; Supplementary Fig. S10B). No significant body weight loss was observed with any of the treatments (Fig. 7C; Supplementary Fig. S10C). These results indicate that simultaneous inhibition of all class IA PI3K isoforms strongly induced apoptosis and exhibited significantly greater antitumor efficacy compared with the inhibition of any single class IA PI3K isoform in TRS xenografts, providing proof that pan-class IA inhibition is effective *in vitro* and *in vivo*.

## Discussion

As previously mentioned, the class IA PI3K isoform that primarily functions differs among tumor types, i.e., PI3Kα in *PIK3CA*-mutant carcinomas, PI3Kβ in *PTEN*-deficient prostate cancers, and PI3Kδ in hematologic malignancies such as CLL, SLL, and FL (9). Therefore, the PI3Kα-specific inhibitor alpelisib and several PI3Kδ-specific inhibitors, including idelalisib, have been approved by the US FDA for the treatment of patients with *PIK3CA*-mutated ER^+^HER2^−^ breast cancers and the aforementioned B-cell malignancies, respectively (25, 29). In the present study, we clearly demonstrated that the PI3Kα isoform primarily functions in TRS cells and in carcinoma cells harboring a *PIK3CA* mutation; however, unlike *PIK3CA*-mutant carcinoma cells, PI3Kβ and PI3Kδ can complement the function of PI3Kα to activate the PI3K pathway, which seems to be a characteristic feature of TRS cells. Therefore, inhibition of PI3Kβ and PI3Kδ in addition to PI3Kα was required to sufficiently suppress PI3K downstream signaling, thereby inhibiting proliferation and ultimately inducing apoptosis in TRS cells. These observations provide proof that triple inhibition of class IA PI3K isoforms using pan-PI3K inhibitors, including ZSTK474, could serve as a promising therapeutic strategy for the treatment of TRS.

One possible explanation for the strong effect of combined triple inhibition of class IA PI3K isoforms is that the proliferation and survival of TRS cells following PI3Kα inhibition become highly dependent on residual PI3K signaling mediated by PI3Kβ and PI3Kδ. Inactivation of all class IA PI3K isoforms led to marked growth inhibition and induction of apoptosis in hematopoietic progenitor cells and mouse embryonic fibroblasts (33). Importantly, even if approximately 90% of total class IA PI3K activity was suppressed by coinactivation of PI3Kα and PI3Kδ, the remaining PI3K activity conferred by PI3Kβ can maintain growth and survival in these cells (33). Similarly, our results illustrated that although inhibition of PI3Kα alone almost completely suppressed PI3K signaling activity, resulting in a low level of phosphorylated Akt, only partial inhibition of proliferation and induction of apoptosis were achieved in TRS cells. In fact, combinatorial inhibition of PI3Kα with PI3Kβ and/or PI3Kδ suppressed the residual PI3K signaling activity, leading to strong inhibition of proliferation and induction of apoptosis. This highlights the significant roles of PI3Kβ and PI3Kδ in maintaining minimal PI3K signaling activity upon PI3Kα inhibition, which is crucial for the proliferation and survival of TRS cells.

The mechanism of compensatory PI3K signaling by PI3Kβ and PI3Kδ in the absence of PI3Kα activity remains unclear. One plausible mechanism is that inhibition of PI3Kα relieves negative feedback of RTK signaling, which in turn activates other class IA PI3K isoforms to restore the activity of PI3K downstream signaling via Akt and mTOR, leading to resistance to PI3Kα inhibition, as reported in *PIK3CA*-mutant or *HER2*-amplified luminal breast cancers (34, 35). Likewise, upon PI3Kβ inhibition, activation of PI3Kα because of the relief of the negative feedback of RTKs, androgen receptor, and estrogen receptor recovered the activity of PI3K downstream signaling, leading to resistance to PI3Kβ inhibition in *PTEN*-mutated tumors (36). We previously reported that the negative feedback of IGF1R caused by serine phosphorylation of insulin receptor substrate 1 via p70S6K is relieved upon inhibition of class IA PI3K isoforms, which reactivates PI3K downstream signaling, leading to resistance to PI3K inhibitors in several tumor types (37, 38). Therefore, in the present study, the residual PI3K downstream signaling activity observed upon PI3Kα inhibition in TRS cells might arise from the activation of PI3Kβ and/or PI3Kδ through the relief of negative feedback of RTKs such as IGF1R, thereby maintaining proliferation and survival. Phosphoproteomic analysis revealed that the pan-PI3K inhibitor ZSTK474 had a greater effect on the Akt/mTOR pathway than alpelisib, further supporting the hypothesis that PI3Kα inhibition caused reactivation of PI3K downstream signaling in TRS cells.

Another finding from phosphoproteomic analysis was that casein kinase 2 α 1, the gene encoding CK2α, was more strongly suppressed by ZSTK474 than by alpelisib. CK2 is known to activate PI3K signaling via PTEN and Akt phosphorylation and contribute to the inhibition of apoptosis via caspase-3 phosphorylation (39). Previous studies reported that inhibition of both CK2 and PI3K signaling synergistically induces apoptosis in acute myeloid leukemia and colon cancer (40). There are two possible explanations for CK2 inhibition upon ZSTK474 treatment. One is that CK2 is directly inhibited by ZSTK474. However, we previously reported that the inhibitory effects of ZSTK474 on CK2 and other protein kinases are relatively weak, which excludes this possibility. The other is that CK2 acts downstream of PI3Kβ and PI3Kδ, and their inhibition results in CK2 inhibition. As previously mentioned, inhibition of PI3Kβ and PI3Kδ in combination with PI3Kα completely suppressed the residual activity of PI3K downstream signaling, as determined by immunoblotting of phosphorylated Akt. This suggests that all three isoforms likely regulate canonical PI3K downstream signaling including Akt and mTOR in common. Therefore, it seems unlikely that PI3Kβ and PI3Kδ regulate distinct downstream signaling from PI3Kα. Further studies are needed to elucidate both the involvement of CK2 in the downstream signaling of PI3Kβ/δ and its role in the antitumor effect of pan-PI3K inhibitors.

The upstream signaling of PI3Kβ/δ isoforms involved in the proliferation and survival of TRS cells remains unclear. In general, RTK downstream signaling in response to growth factor stimuli primarily uses PI3Kα, but PI3Kβ and PI3Kδ are also utilized. In Ewing sarcoma harboring the *EWSR1::FLI1* fusion gene, the IGF1R pathway is known to be activated, and it is considered a promising therapeutic target (41–43). Conversely, PDGFRα and c-MET is commonly activated in SS with the *SS18::SSX* fusion gene (44), whereas c-MET is also activated in clear cell sarcomas harboring the *EWSR1::ATF1* fusion gene (45). Typically, PI3Kδ is found primarily in leukocytes, and it mediates the signals of immune receptors such as T-cell antigen receptor (TCR), BCR, and cytokine receptors (9). Previously, we and other groups reported that PI3Kδ mediates the signals from TCR and cytokine receptors in T cells and regulates the activation of tumor-infiltrating regulatory T cells and differentiation of memory T cells (16, 46). However, in the present study, we detected p110δ expression in TRS cells and the significant contribution of PI3Kδ to the proliferation and survival of TRS cells upon PI3Kα inhibition. Likewise, PI3Kδ has been reported to contribute to the proliferation and survival of several solid tumors such as neuroblastoma (47), hepatocellular carcinoma (48), and colon cancer (49). In addition, RTKs such as EGFR and IGF1R have been reported to be upstream receptors of PI3Kδ in neuroblastoma (47). Similarly, as reported in neuroblastoma, PI3Kδ might be activated downstream of activated RTKs in TRSs. Other than growth factors and their receptors, G protein–coupled receptors (GPCR) might also participate in the upstream event of PI3K activation in TRS cells, in which case PI3Kβ is more likely to be involved than PI3Kα and PI3Kδ. Reportedly, lysophosphatidic acid/lysophosphatidic acid receptor and C-X-C motif chemokine ligand 12/C-X-C motif chemokine receptor 4 are involved in the proliferation and survival of osteosarcoma and malignant peripheral nerve sheath tumor, respectively, through activation of the PI3K/Akt pathway (50). Therefore, some unidentified GPCR or RTK signaling pathway might explain the involvement of PI3Kβ and PI3Kδ in TRS cells, which should be identified in our future studies.

In the clinical development of PI3K inhibitors for sarcomas, including TRSs, biomarkers that reflect target engagement and therapeutic efficacy are important. In the present study, phosphorylation levels of Akt (T308 and S473) and S6 (S235/236) in TRSs following PI3K inhibition correlated well with antitumor efficacy. Consistent downregulation of phosphorylated Akt (S473) and S6 (S235/236) in tumor cells following ZSTK474 treatment has been observed in a phase Ib clinical trial (13). In addition, apoptosis-related proteins, including cleaved PARP, cleaved caspase-3, PUMA, and BIM, were also closely associated with drug efficacy, which we have previously reported (14, 15). These molecules may serve as clinically relevant pharmacodynamic biomarkers for monitoring PI3K pathway inhibition and therapeutic response in sarcomas. Another important consideration in the clinical application of PI3K inhibitors is toxicity, particularly hyperglycemia. As Akt2 is known to play a major role in glucose metabolism, particularly in metabolic tissues such as the liver and skeletal muscle, evaluation of Akt2 phosphorylation in metabolic tissues upon PI3K inhibitor treatment is important. In this study, we examined phosphorylation of Akt at T308 and S473 in TRS cells using an antibody that detects all Akt isoforms (Akt1-3). However, we did not specifically evaluate the phosphorylation status of Akt2 in metabolic tissues. The effects of our PI3K inhibitor on phosphorylation of Akt2 and blood glucose levels will need to be evaluated in future studies.

In summary, PI3K signaling and consequent proliferation and survival in TRS cells were predominantly regulated by PI3Kα, with only minor contributions from PI3Kβ, PI3Kδ, and PI3Kγ. However, when PI3Kα was inhibited, PI3K signaling, proliferation, and survival in TRS cells became highly dependent on PI3Kβ and PI3Kδ, and the combinatorial inhibition of PI3Kα, PI3Kβ, and PI3Kδ fully potentiated the antitumor activity and eventually induced apoptosis. Although it remains unclear why this happens selectively in TRS cells, pan-class IA PI3K inhibitors are expected to be more effective against TRSs than in other tumor types such as carcinomas and lymphomas. Although the results from initial clinical trials testing the use of pan-PI3K inhibitors for solid tumors have been disappointing (10), our current findings provide a rationale for developing sarcoma therapies with pan-PI3K inhibitors.

## Supplementary Material

Supplementary Table S1 Antibodies used in this study

Supplementary Table S2 Mutations of RTK and TP53 genes in sarcoma cell lines

Supplementary Fig. S1 PI3K pathway genetic alterations in clinical TRS samples

Supplementary Fig. S2 Correlation of mRNA and protein expression of class I PI3K isoforms in sarcoma cell lines

Supplementary Fig. S3 Expression of PI3K pathway genes in sarcoma cell lines and clinical samples

Supplementary Fig. S4 PI3K isoform-specific inhibition by selective inhibitors

Supplementary Fig. S5 Growth inhibition of TRS cell lines by PI3K inhibitors

Supplementary Fig. S6 Simultaneous inhibition of PI3Kα with PI3Kβ/δ induces synergistic antitumor effects and apoptosis in SJCRH30 but not MKN1 cells

Supplementary Fig. S7 Simultaneous inhibition of PI3Kα with PI3Kβ/δ enhances suppression of cell growth and cell cycle in SJCRH30 but not MKN1 cells

Supplementary Fig. S8 Simultaneous inhibition of PI3Kα with PI3Kβ/δ enhances suppression of cell growth without inducing apoptosis in non-sarcoma cancer cell lines

Supplementary Fig. S9 Simultaneous inhibition of PI3Kα with PI3Kβ/δ induces significant apoptosis associated with suppression of Akt/mTOR signaling

Supplementary Fig. S10 Simultaneous inhibition of PI3Kα with PI3Kβ/δ suppresses tumor growth with PI3K pathway inhibition and apoptosis induction in the SYO-1 xenograft model

## Data Availability

The raw whole-exome sequencing and RNA-seq data have been deposited in the Sequence Read Archive database (RRID: SCR_001370) under accession number PRJNA1242479. The raw proteomic data has been deposited in the ProteomeXchange Consortium via the jPOST repository under accession number PXD062232. RNA-seq data of SU-DHL-4 cells (GSM6285348), SU-DHL-6 cells (GSM6285350), SS (GSM8378222, GSM8378226, GSM8378227, GSM8378248, and GSM8378286), Ewing sarcoma (GSM8332627-GSM8332631), breast cancer (GSM8141778-GSM8141782), and DLBCL (GSM5402206-GSM5402210) were obtained from the GEO database. All other raw data are available upon request to the corresponding author.
