# Supplementary material for: Cooperative Roles of Class IA PI3K Isoforms in Translocation-Related Sarcoma Cell Survival and Proliferation
Source: Cancer Res Commun. 2026 Apr 29;6(4):976–93. doi: 10.1158/2767-9764.CRC-25-0787 (PMC13127112; doi:10.1158/2767-9764.CRC-25-0787)
Supplement: Supplementary Fig. S1 — PI3K pathway genetic alterations in clinical TRS samples [file crc-25-0787_supplementary_fig.s1_suppsf1.pdf]

Supplementary Fig. S1

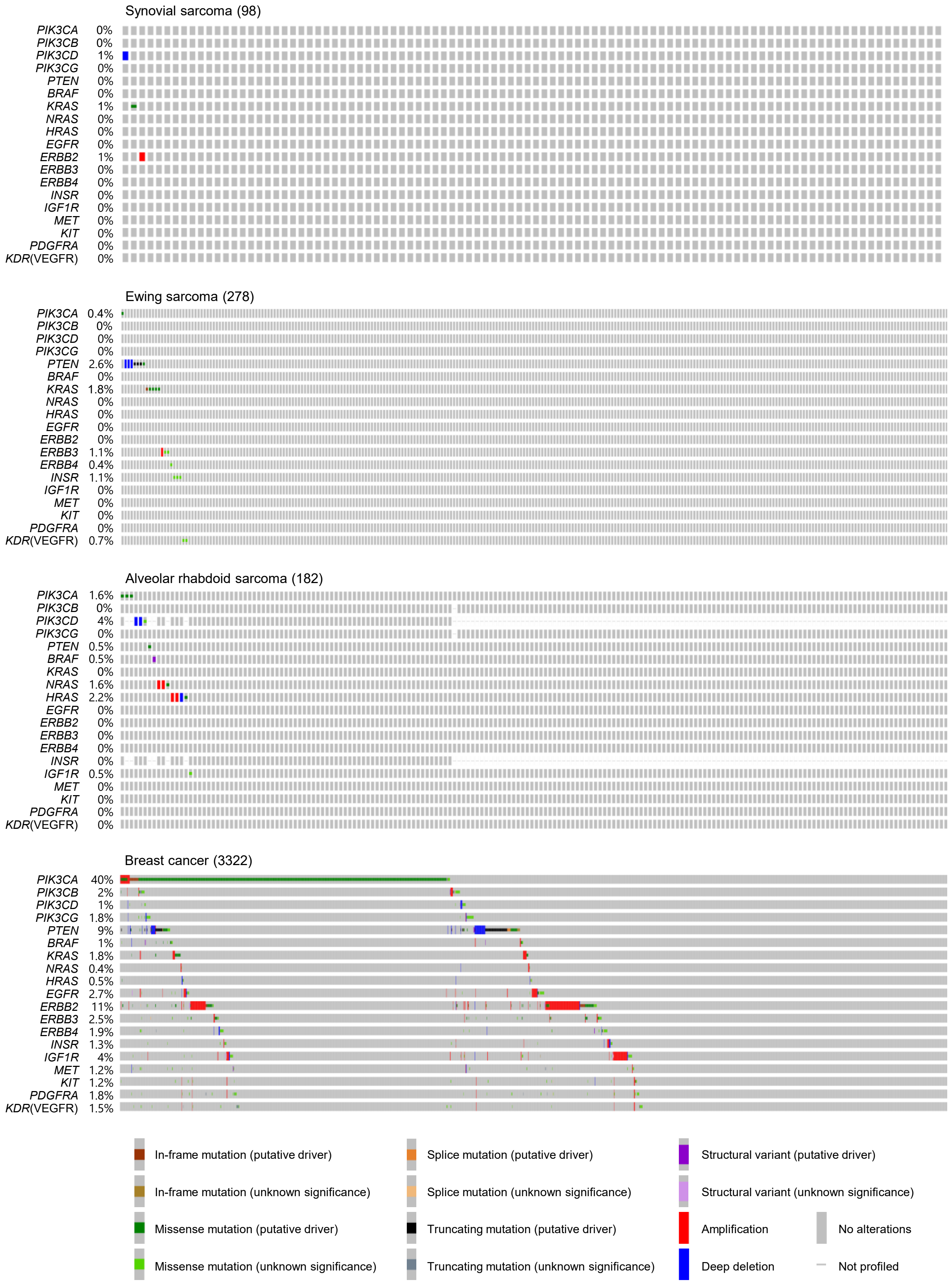

Supplementary Fig. S1. The genetic alterations of PI3K isoforms and PI3K-related genes in clinical samples of TRSs and breast cancer.
Each column represents a sample. The number of samples is indicated in parentheses.
The alteration frequencies of each gene are shown to the right of the gene names.
