# Supplementary material for: Cooperative Roles of Class IA PI3K Isoforms in Translocation-Related Sarcoma Cell Survival and Proliferation
Source: Cancer Res Commun. 2026 Apr 29;6(4):976–93. doi: 10.1158/2767-9764.CRC-25-0787 (PMC13127112; doi:10.1158/2767-9764.CRC-25-0787)
Supplement: Supplementary Fig. S2 — Correlation of mRNA and protein expression of class I PI3K isoforms in sarcoma cell lines [file crc-25-0787_supplementary_fig.s2_suppsf2.pdf]

Supplementary Fig. S2

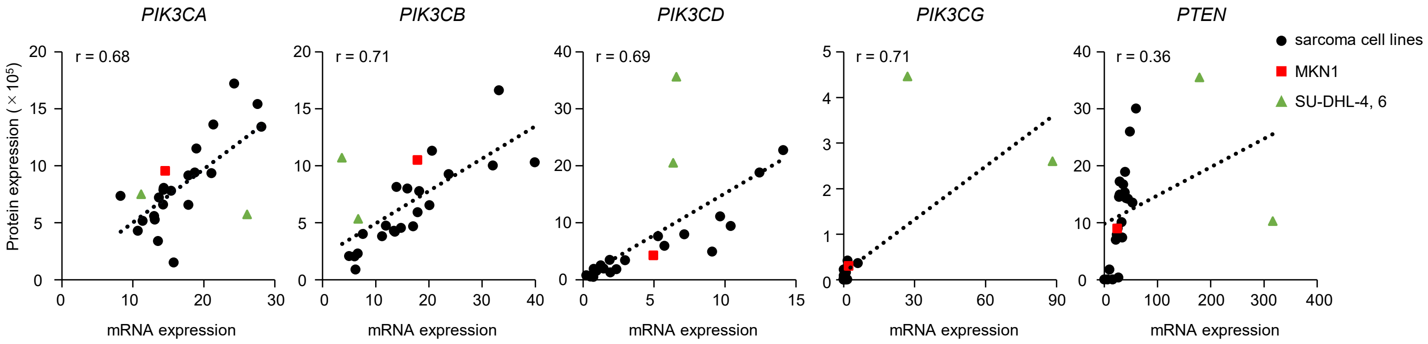

Supplementary Fig. S2. Correlation analysis of the mRNA and protein expression of class I PI3K isoforms and *PTEN* in sarcoma cell lines

Dot plots presenting the correlation between the mRNA and protein expression of the catalytic subunits of class I PI3K isoforms (*PIK3CA, PI3KCB, PI3KCD, PI3KCG*) and *PTEN* in sarcoma cell lines, MKN1, SU-DHL-4, and SU-DHL-6 cells by RNA-seq and immunoblotting as presented in **Fig. 1A and B.**
