# Supplementary material for: Cooperative Roles of Class IA PI3K Isoforms in Translocation-Related Sarcoma Cell Survival and Proliferation
Source: Cancer Res Commun. 2026 Apr 29;6(4):976–93. doi: 10.1158/2767-9764.CRC-25-0787 (PMC13127112; doi:10.1158/2767-9764.CRC-25-0787)
Supplement: Supplementary Fig. S3 — Expression of PI3K pathway genes in sarcoma cell lines and clinical samples [file crc-25-0787_supplementary_fig.s3_suppsf3.pdf]

Supplementary Fig. S3

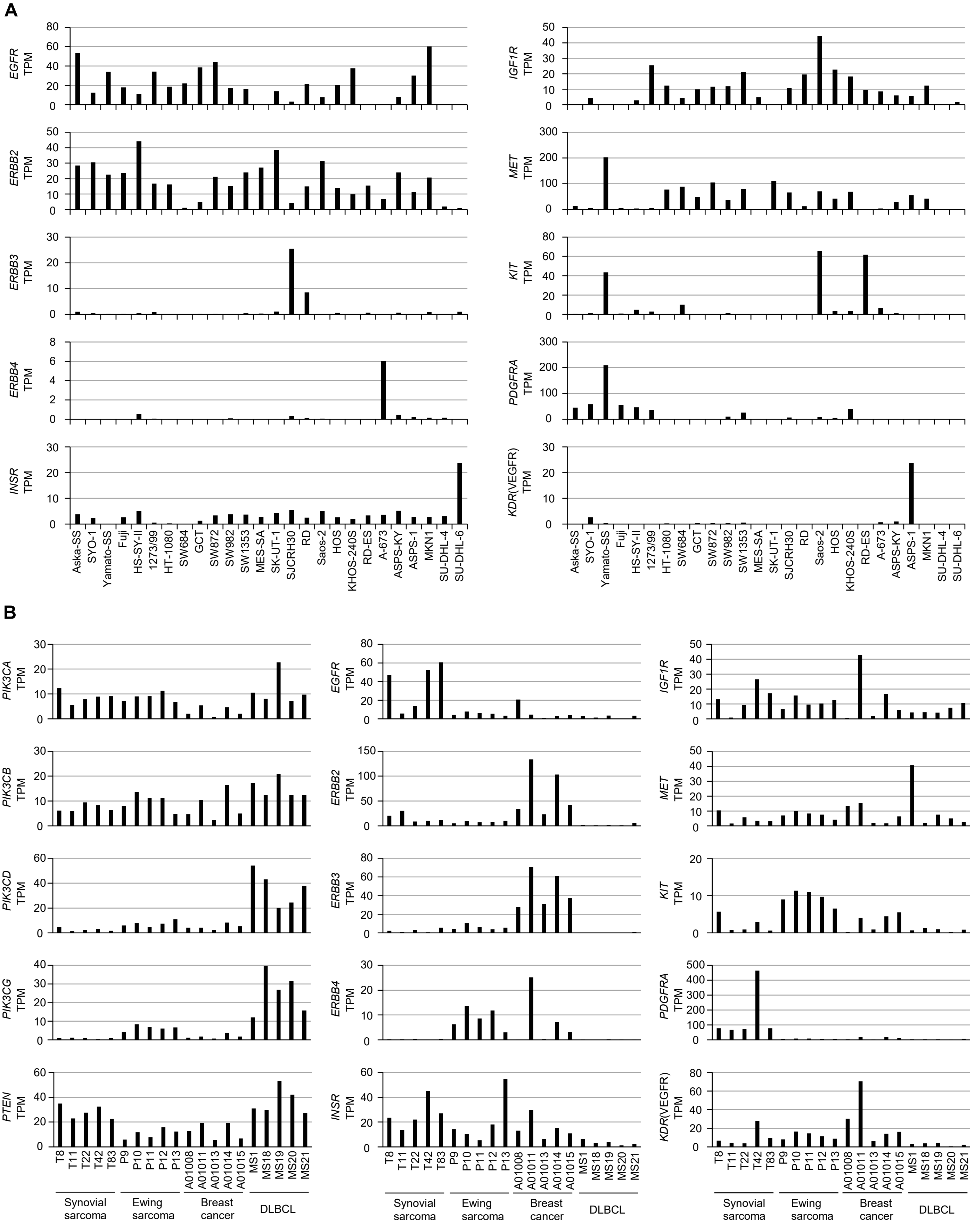

Supplementary Fig. S3. The expression of PI3K isoforms and PI3K-related genes in sarcoma cell lines and clinical samples.

A, The expression of the indicated RTK genes in the indicated sarcoma cell lines, a gastric cancer cell line, and diffuse large B-cell lymphoma cell lines determined by RNA-seq. **B,** The expression of the indicated PI3K isoform and PI3K-related genes in synovial sarcoma, Ewing sarcoma, breast cancer and DLBCL clinical samples determined by RNA-seq.
