# Supplementary material for: Cooperative Roles of Class IA PI3K Isoforms in Translocation-Related Sarcoma Cell Survival and Proliferation
Source: Cancer Res Commun. 2026 Apr 29;6(4):976–93. doi: 10.1158/2767-9764.CRC-25-0787 (PMC13127112; doi:10.1158/2767-9764.CRC-25-0787)
Supplement: Supplementary Fig. S4 — PI3K isoform-specific inhibition by selective inhibitors [file crc-25-0787_supplementary_fig.s4_suppsf4.pdf]

Supplementary Fig. S4

A

Inhibitor (IC50, nmol/L)	PI3Kα	PI3Kβ	PI3Kδ	Reference
**Alpelisib**	4.6	1156	290	22
**TGX-221**	5000	5	100	23
**Idelalisib**	1089	664	7	24
**ZSTK474**	16	44	4.6	25

B

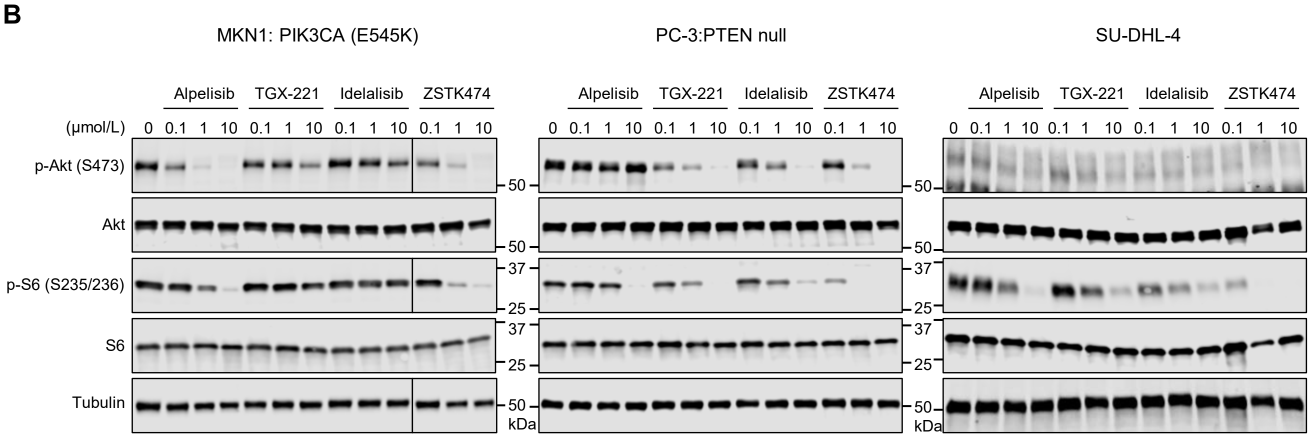

C

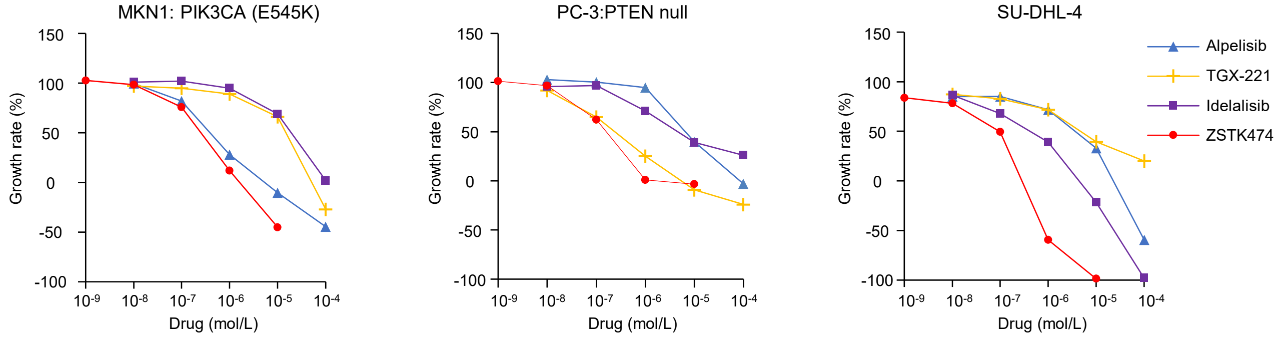

Supplementary Fig. S4. Alpelisib, TGX-221, idelalisib, and ZSTK474 inhibit PI3Kα, PI3Kβ, PI3Kδ, and PI3Kα/β/δ, respectively, at comparable concentrations at the biochemical and cellular levels

A, Efficacy of PI3K inhibitors against PI3K isoform activity at the biochemical level. **B,** Immunoblots of the indicated proteins in MKN1 gastric cancer cells carrying the *PIK3CA* hotspot mutation E545K, PC-3 prostate cancer cells with *PTEN* loss, and SU-DHL-4 diffuse large B-cell lymphoma cells treated with the indicated drugs for 48 hours. Tubulin was used as a loading control. **C,** Concentration–response curves of alpelisib, TGX-221, idelalisib, and ZSTK474 in the indicated cell lines.
