# Supplementary material for: Cooperative Roles of Class IA PI3K Isoforms in Translocation-Related Sarcoma Cell Survival and Proliferation
Source: Cancer Res Commun. 2026 Apr 29;6(4):976–93. doi: 10.1158/2767-9764.CRC-25-0787 (PMC13127112; doi:10.1158/2767-9764.CRC-25-0787)
Supplement: Supplementary Fig. S5 — Growth inhibition of TRS cell lines by PI3K inhibitors [file crc-25-0787_supplementary_fig.s5_suppsf5.pdf]

Supplementary Fig. S5

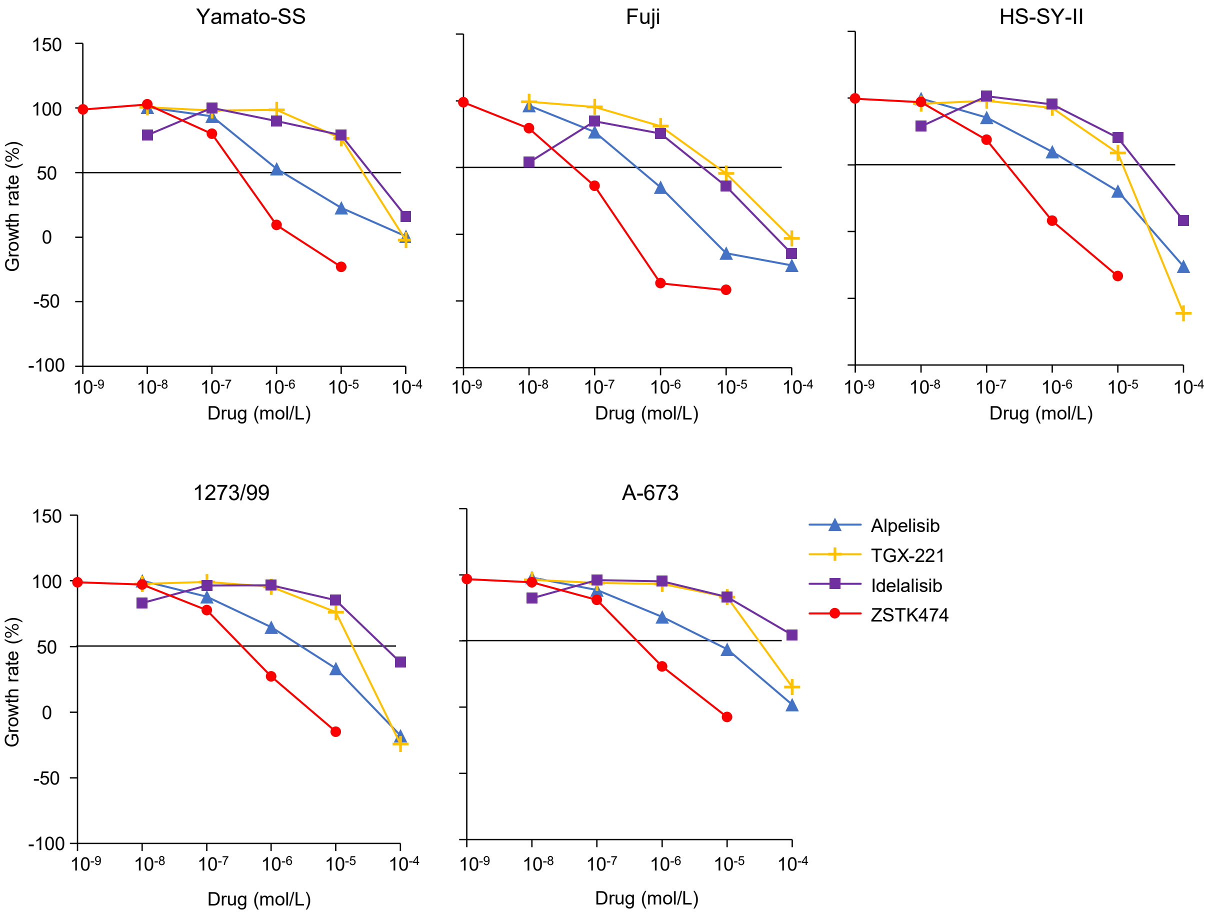

Supplementary Fig. S5. The *in vitro* growth inhibitory effects of alpelisib, TGX-221, idelalisib, and ZSTK474 against TRS cell lines.
Concentration–response curves of alpelisib, TGX-221, idelalisib, and ZSTK474 in the indicated TRS cell lines.
