# Supplementary material for: Cooperative Roles of Class IA PI3K Isoforms in Translocation-Related Sarcoma Cell Survival and Proliferation
Source: Cancer Res Commun. 2026 Apr 29;6(4):976–93. doi: 10.1158/2767-9764.CRC-25-0787 (PMC13127112; doi:10.1158/2767-9764.CRC-25-0787)
Supplement: Supplementary Fig. S6 — Simultaneous inhibition of PI3Kα with PI3Kβ/δ induces synergistic antitumor effects and apoptosis in SJCRH30 but not MKN1 cells [file crc-25-0787_supplementary_fig.s6_suppsf6.pdf]

Supplementary Fig. S6

A

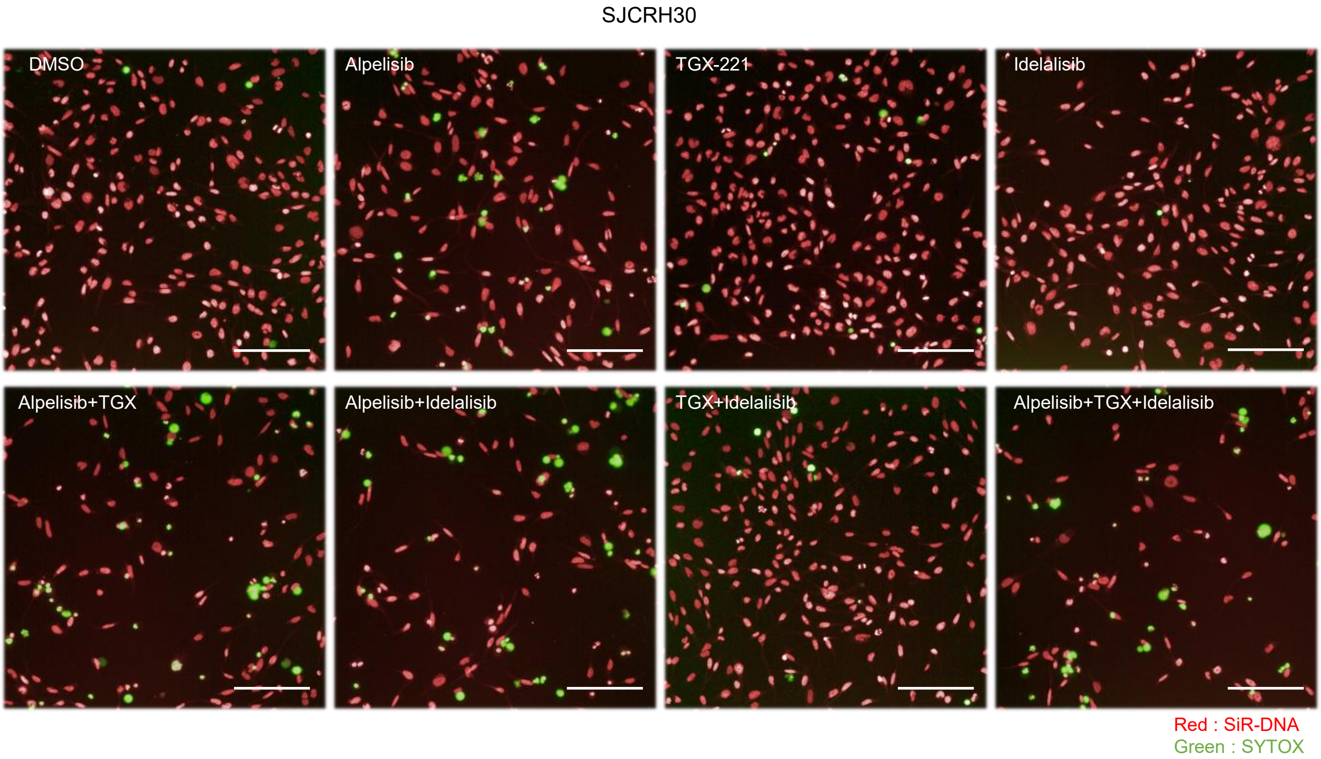

B

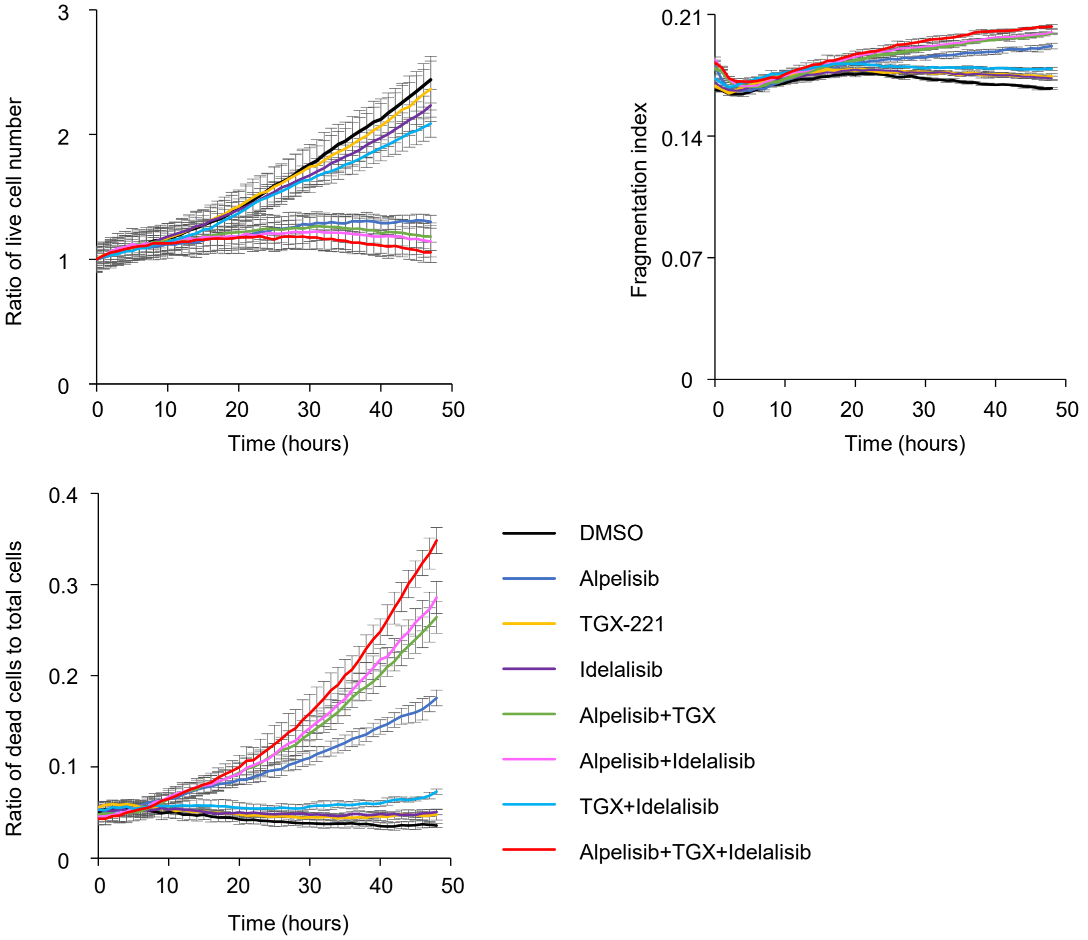

Supplementary Fig. S6 (continued)

C

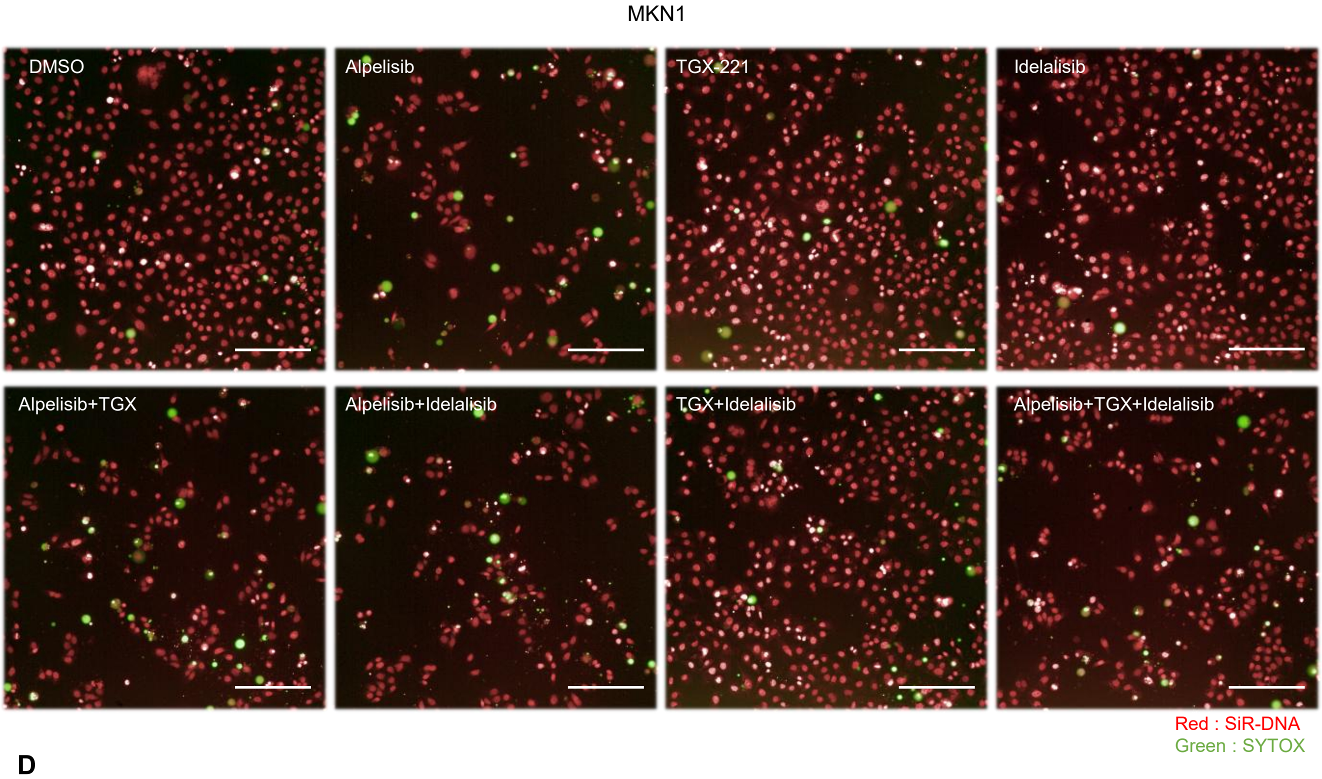

D

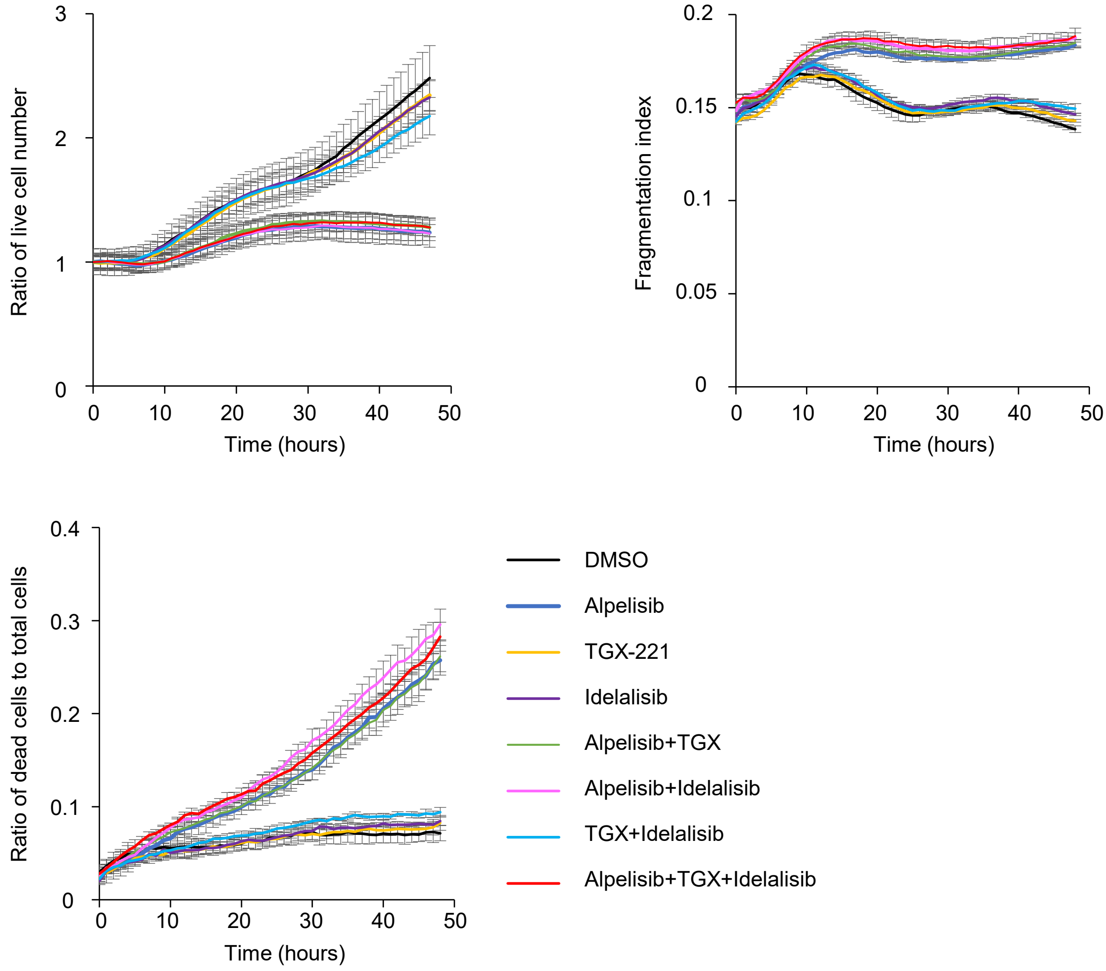

Supplementary Fig. S6. Simultaneous inhibition of PI3Kα with PI3Kβ and/or PI3Kδ results in synergistic antitumor effects and apoptosis induction in SJCRH30, but not in MKN1 cells

A-D, Fluorescence time-lapse imaging analysis in SJCRH30 ARMS cells carrying the fusion gene *PAX3::FOXO1* (**A, B**) and MKN1 gastric cancer cells carrying the *PIK3CA* hotspot mutation E545K (**C, D**) stained with SiR-DNA and SYTOX Green followed by treatment with alpelisib, TGX-221, and idelalisib alone or in combination at 8 μmol/L for 48 hours. Representative images of SJCRH30 (**A**) and MKN1 cells (**C**) at 48 h are presented. The growth rate of live cells (top left), the fragmentation index (top right), and the proportion of dead cells to total cells (bottom) in SJCRH30 (**B**) and MKN1 cells (**D**) were calculated by quantitative image analysis. Data are presented as the mean ± SD (n = 5).
