# Supplementary material for: Cooperative Roles of Class IA PI3K Isoforms in Translocation-Related Sarcoma Cell Survival and Proliferation
Source: Cancer Res Commun. 2026 Apr 29;6(4):976–93. doi: 10.1158/2767-9764.CRC-25-0787 (PMC13127112; doi:10.1158/2767-9764.CRC-25-0787)
Supplement: Supplementary Fig. S7 — Simultaneous inhibition of PI3Kα with PI3Kβ/δ enhances suppression of cell growth and cell cycle in SJCRH30 but not MKN1 cells [file crc-25-0787_supplementary_fig.s7_suppsf7.pdf]

Supplementary Fig. S7

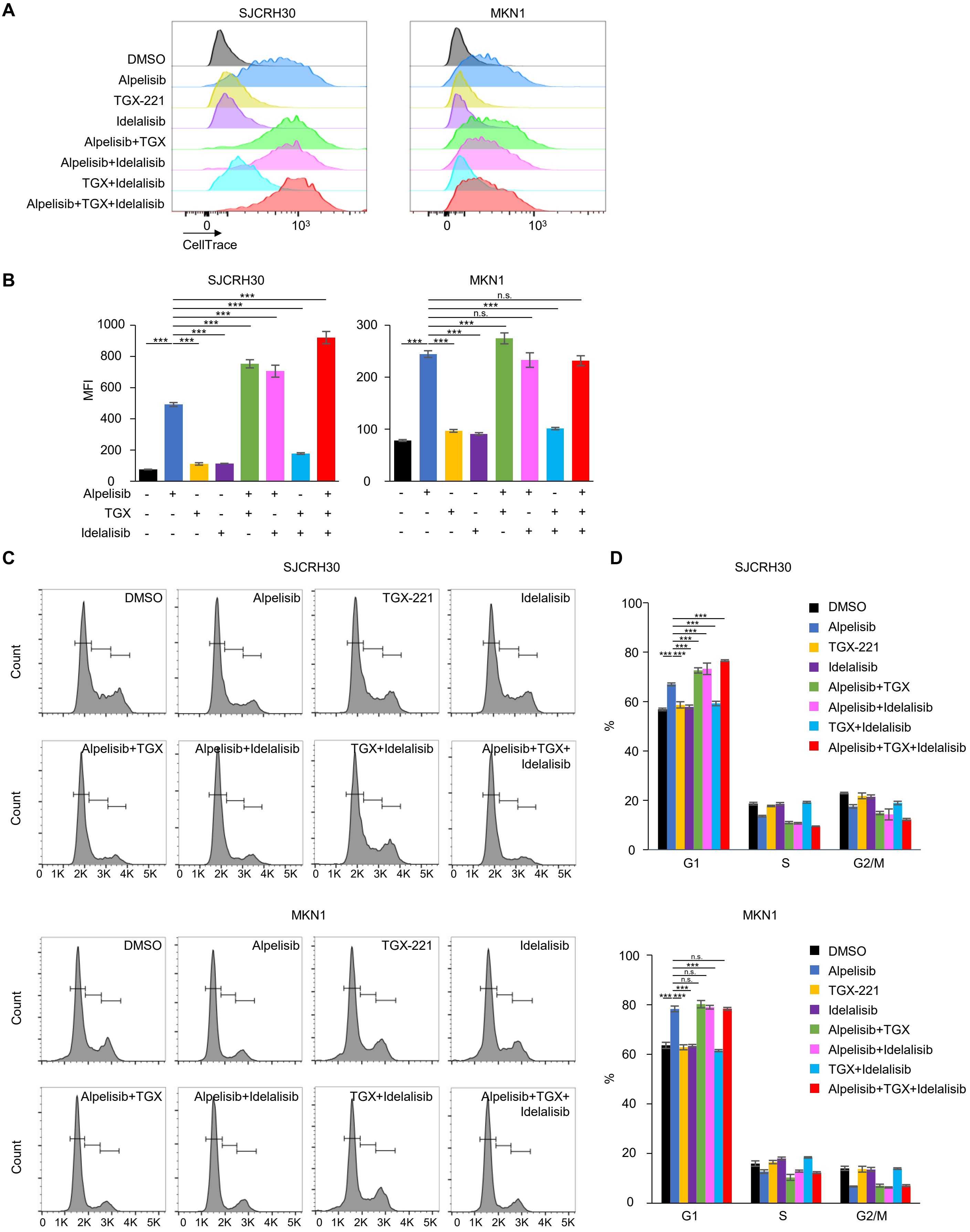

Supplementary Fig. S7. Simultaneous inhibition of PI3Kα with PI3Kβ and/or PI3Kδ augments the suppression of proliferation and cell cycle progression in SJCRH30, but not in MKN1 cells

A, Representative flow cytometry histograms of the proliferation of SJCRH30 or MKN1 cells stained with CellTracer Yellow and treated with alpelisib, TGX-221, and idelalisib alone or in combination at 8 μmol/L. Proliferation was examined by CellTracer Yellow dilution after culture with or without the drugs for 6 days. **B,** Summaries of the median fluorescence intensity (MFI) of CellTracer Yellow in the cell proliferation assay presented in **A**. Data are presented as the mean ± SD (n = 4). Statistical analysis was performed by one-way ANOVA with Dunnett's *post hoc* test. n.s.: not significant, ***$P < 0.001$. **C,** Representative flow cytometry histograms of the cell cycle distribution (G1, S, and G2/M) in SJCRH30 (top) or MKN1 cells (bottom) treated with alpelisib, TGX-221, and idelalisib alone or in combination at 8 μmol/L for 48 hours. **D,** Summaries of the cell cycle distribution (G1, S, and G2/M) presented in **C**. Data are presented as the mean ± SD (n = 4). Statistical analysis was performed by one-way ANOVA with Dunnett's *post hoc* test. n.s.: not significant, ***$P < 0.001$.
