# Supplementary material for: Cooperative Roles of Class IA PI3K Isoforms in Translocation-Related Sarcoma Cell Survival and Proliferation
Source: Cancer Res Commun. 2026 Apr 29;6(4):976–93. doi: 10.1158/2767-9764.CRC-25-0787 (PMC13127112; doi:10.1158/2767-9764.CRC-25-0787)
Supplement: Supplementary Fig. S8 — Simultaneous inhibition of PI3Kα with PI3Kβ/δ enhances suppression of cell growth without inducing apoptosis in non-sarcoma cancer cell lines [file crc-25-0787_supplementary_fig.s8_suppsf8.pdf]

Supplementary Fig. S8

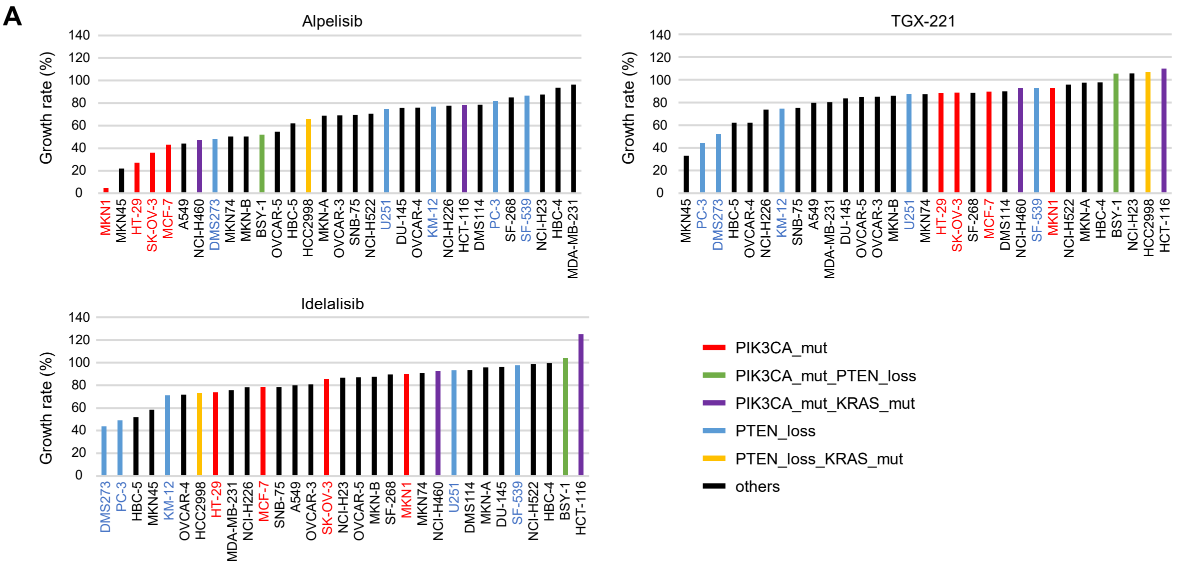

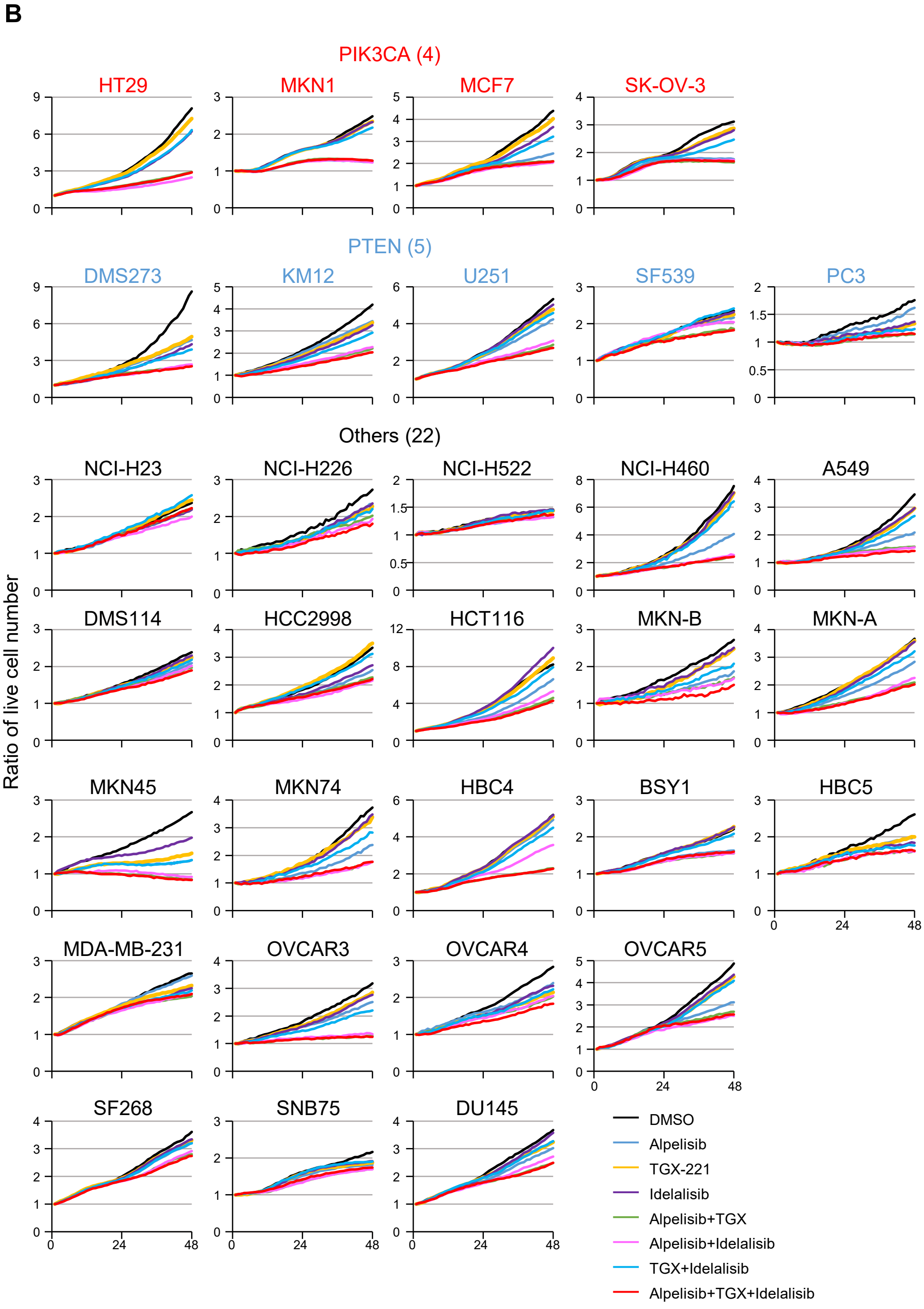

PIK3CA	Alp+TGX	Alp+Ide	Alp+TGX+Ide
HT-29	-12.0	-20.3	-37.8
MKN1	-9.1	-6.7	-18.0
MCF-7	-0.1	-9.6	-21.5
SK-OV-3	-6.2	-12.9	-22.7

PTEN	Alp+TGX	Alp+Ide	Alp+TGX+Ide
DMS273	-20.6	-30.2	-76.5
KM-12	13.3	7.6	-10.3
U251	18.8	19.7	15.9
SF-539	15.1	6.6	15.5
PC-3	7.3	-0.4	-45.2

Others	Alp+TGX	Alp+Ide	Alp+TGX+Ide
NCI-H23	-1.7	1.5	-15.3
NCI-H226	-7.6	2.2	-16.6
NCI-H522	-14.4	-2.2	-15.0
NCI-H460	17.1	16.1	10.5
A549	0.5	1.9	-13.3
DMS114	-1.2	-1.1	-2.3
HCC2998	11.3	-10.9	-13.2
HCT-116	28.0	18.1	32.4
MKN-B	-4.8	-1.2	-5.4
MKN-A	25.4	17.7	23.8
MKN45	-35.6	-14.3	-76.1
MKN74	9.7	12.8	0.7
HBC-4	60.3	32.1	60.6
BSY-1	6.4	5.9	2.5
HBC-5	-15.0	-23.2	-62.1
MDA-MB-231	13.8	5.6	-13.8
OVCAR-3	41.3	34.1	23.9
OVCAR-4	-17.8	-9.1	-35.4
OVCAR-5	-4.3	2.6	-14.2
SF-268	4.5	1.4	-3.4
SNB-75	-15.7	-11.7	-40.7
DU-145	3.7	7.7	-0.2

TRS	Alp+TGX	Alp+Ide	Alp+TGX+Ide
ASKA	48.9	35.7	45.8
SYO1	15.7	7.5	4.6
SJCRH30	3.1	-3.4	-2.5

Supplementary Fig. S8 (continued)

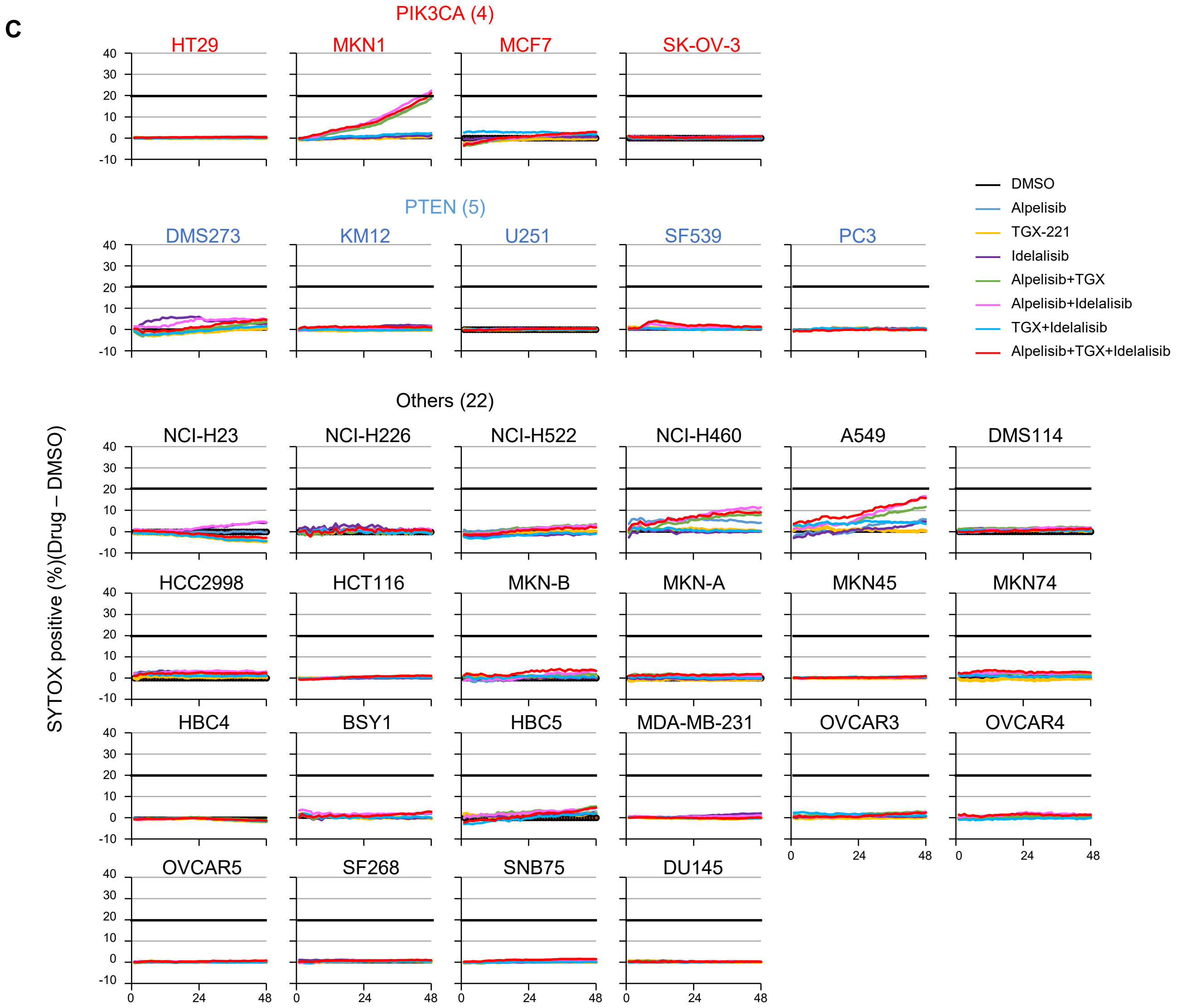

Supplementary Fig. S8. Simultaneous inhibition of PI3Kα with PI3Kβ and/or PI3Kδ enhances growth inhibition but does not induce apoptosis in diverse non-sarcoma cancer cell lines

A, Growth rate (%) of non-sarcoma cell lines treated with the indicated drugs at 8 μmol/L for 48 hours determined by fluorescence time-lapse imaging analysis. **B,** Plots showing growth rate of non-sarcoma cell lines treated with the indicated drugs at 8 μmol/L. The right panels show the Combination Score (CS) for the indicated combination treatments. The CS is calculated as the growth inhibition rate (%) (GI: 100 – Growth rate (%)) of each combination treatment minus the sum of the GI of the individual drugs used in that combination. An example of the formula is shown below.

$CS_{Alpelisib+TGX+Idelalisib} = GI_{Alpelisib+TGX+Idelalisib} – (GI_{Alpelisib} + GI_{TGX} + GI_{Idelalisib})$

GI of a single agent was set to zero if the GI was negative. **C,** Plots showing SYTOX-positive (%) in non-sarcoma cell lines treated with the indicated drugs at 8 μmol/L.
