# Supplementary material for: Cooperative Roles of Class IA PI3K Isoforms in Translocation-Related Sarcoma Cell Survival and Proliferation
Source: Cancer Res Commun. 2026 Apr 29;6(4):976–93. doi: 10.1158/2767-9764.CRC-25-0787 (PMC13127112; doi:10.1158/2767-9764.CRC-25-0787)
Supplement: Supplementary Fig. S9 — Simultaneous inhibition of PI3Kα with PI3Kβ/δ induces significant apoptosis associated with suppression of Akt/mTOR signaling [file crc-25-0787_supplementary_fig.s9_suppsf9.pdf]

Supplementary Fig. S9

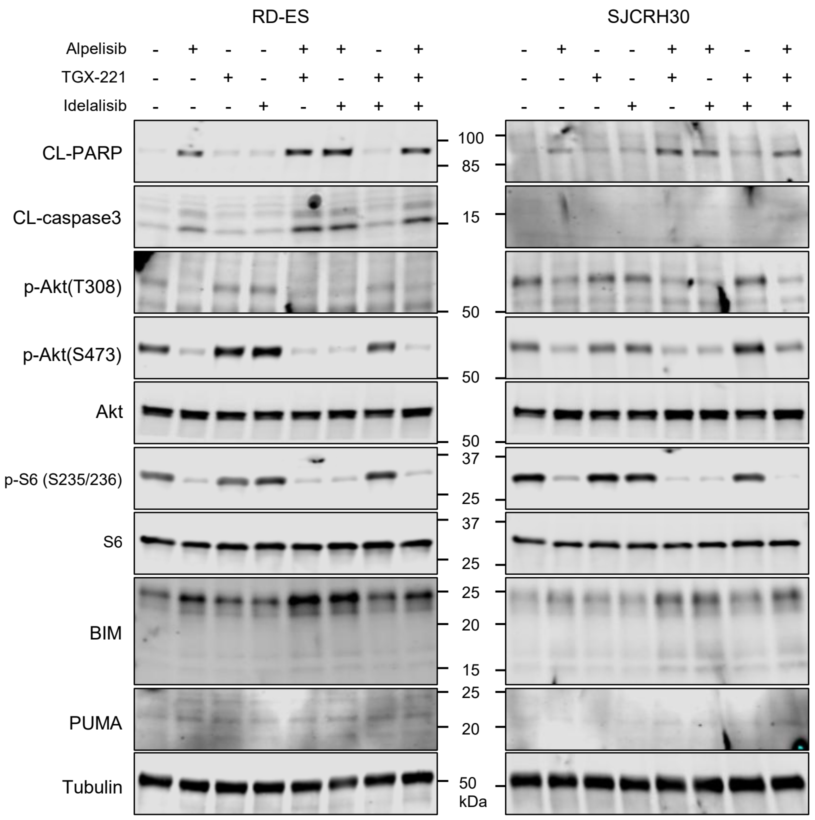

Supplementary Fig. S9. Simultaneous inhibition of PI3Kα with PI3Kβ and/or PI3Kδ results in significant apoptosis induction associated with the suppression of Akt/mTOR signaling Immunoblots of the indicated proteins in RD-ES ES cells carrying the fusion gene *EWSR1::FLI1* and SJCRH30 cells treated with alpelisib, TGX-221, or idelalisib alone or in combination at 1 μmol/L for 48 hours. Tubulin was used as a loading control.
