# Supplementary material for: Cooperative Roles of Class IA PI3K Isoforms in Translocation-Related Sarcoma Cell Survival and Proliferation
Source: Cancer Res Commun. 2026 Apr 29;6(4):976–93. doi: 10.1158/2767-9764.CRC-25-0787 (PMC13127112; doi:10.1158/2767-9764.CRC-25-0787)
Supplement: Supplementary Fig. S10 — Simultaneous inhibition of PI3Kα with PI3Kβ/δ suppresses tumor growth with PI3K pathway inhibition and apoptosis induction in the SYO-1 xenograft model [file crc-25-0787_supplementary_fig.s10_suppsf10.pdf]

Supplementary Fig. S10

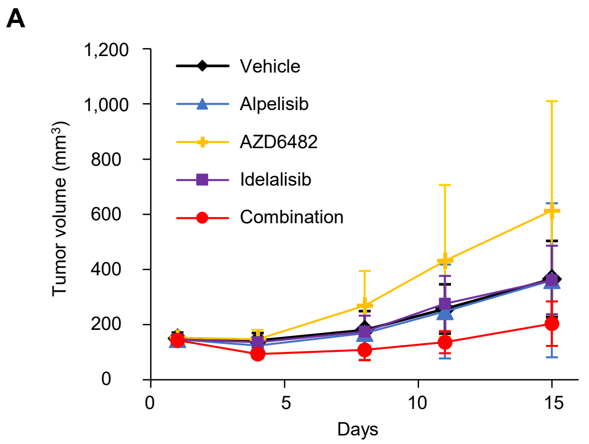


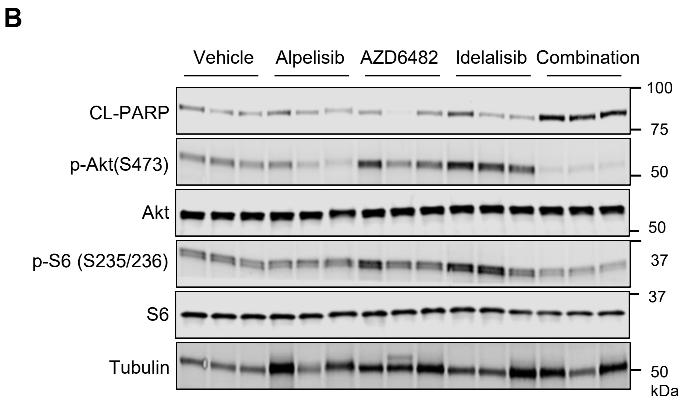


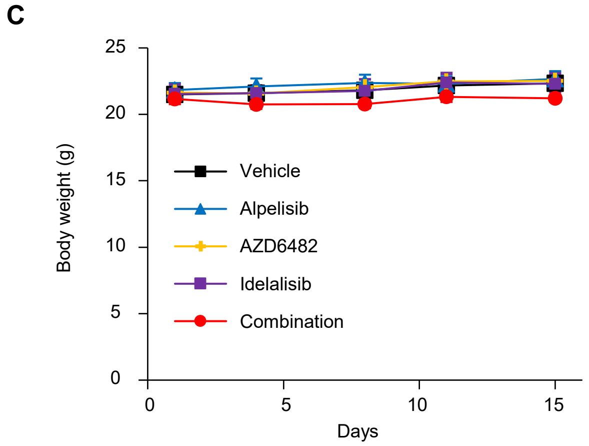


**Supplementary Fig. S10. Simultaneous inhibition of PI3Kα, PI3Kβ, and PI3Kδ leads to significant tumor growth inhibition associated with suppression of PI3K signaling and apoptosis induction in SYO-1 xenograft model**

**A,** Growth curves of SYO-1 xenograft tumors in mice administered alpelisib (10 mg/kg), AZD6482 (20 mg/kg), and idelalisib (30 mg/kg) alone or in combination. Data are presented as the mean ± SD (n = 6). **B,** Immunoblots of the indicated proteins in mice bearing SYO-1 xenograft tumors administered alpelisib, AZD6482, and idelalisib alone or in combination. Tubulin was used as a loading control. **C,** Body wight changes of mice bearing SYO-1 xenograft tumors administered alpelisib, AZD6482, and idelalisib alone or in combination.
